# Bicyclic Schellman Loop Mimics (BSMs): Rigid Synthetic *C*-Caps for Enforcing Peptide Helicity

**DOI:** 10.1021/acscentsci.2c01265

**Published:** 2023-02-13

**Authors:** Tianxiong Mi, Duyen Nguyen, Kevin Burgess

**Affiliations:** Department of Chemistry, Texas A&M University, Box 30012, College Station, Texas 77842, United States

## Abstract

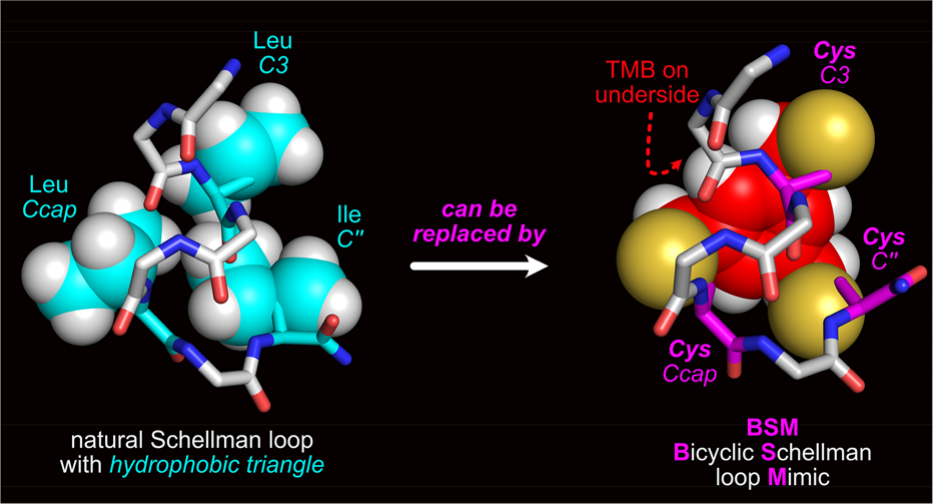

Macrocyclic peptides are the prevalent way to mimic interface helices for disruption of protein interactions, but current strategies to do this via synthetic *C*-cap mimics are underdeveloped and suboptimal. Bioinformatic studies described here were undertaken to better understand Schellman loops, the most common *C*-caps in proteins, to design superior synthetic mimics. An algorithm (Schellman Loop Finder) was developed, and data mining with this led to the discovery that these secondary structures are often stabilized by combinations of three hydrophobic side chains, most frequently from Leu, to form *hydrophobic triangles*. That insight facilitated design of synthetic mimics, bicyclic Schellman loop mimics (BSMs), where the hydrophobic triumvirate was replaced by 1,3,5-trimethylbenzene. We demonstrate that BSMs can be made quickly and efficiently, and are more rigid and helix-inducing than the best current *C*-cap mimics, which are rare and all *mono*cycles.

## Abstract 2

*C*-caps mimics to induce peptide helicity are rare and based on monocyclic strategies. We describe more rigid, helix-enforcing, and easily accessible *bicyclic* designs inspired by a bioinformatic study.

## Introduction

Protein–protein interactions (PPIs) are at the heart of cell signaling pathways.^1^ Chemists can contribute to understanding cell biology, and the biomedicinal consequences of the PPIs, by designing macrocyclic peptides that resemble interface hot segments.^2,3^ Of these, interface helical mimicry is well-studied,^4−6^ and some probes of this type have even progressed to clinical trials.^7,8^

**Figure 1:**
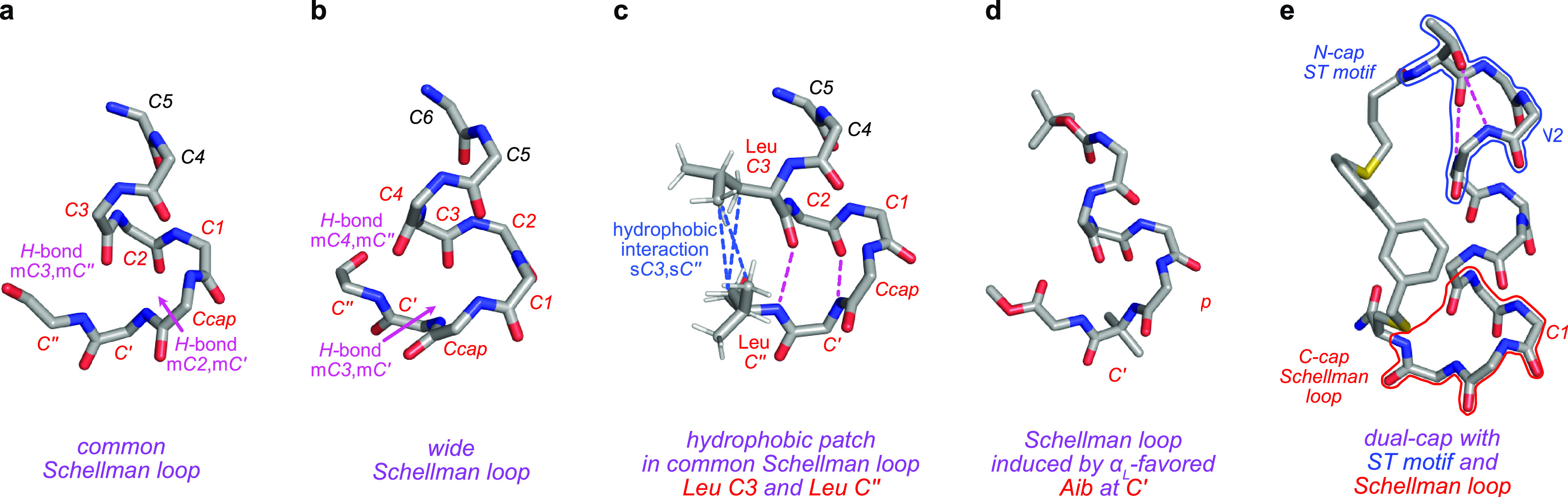
Structural features of Schellman loops and synthetic mimics. (a) Common Schellman loops (from PDB 10MH) have *H*-bonds to the *C*2 and *C*3 carbonyls (m refers to “mainchain” in m*C*3, m*C″*, *etc*.), whereas (b) wide forms (from PDB 1AGX) feature *H*-bonds to the *C*3 and *C*4 carbonyls. (c) Common forms are known to be biased toward forming a *hydrophobic patch* between *C″* and *C*3 side chains (from PDB 1ACO; s refers to “side chain” in s*C*3 and s*C″*). Previously, Schellman loop mimics have been made by (d) incorporating an amino *iso*-butyric acid residue (Aib) at *C′* (from CSD XESNAK)^19,20^ or (e) forming large macrocyclic rings (from PDB 6ANF).

Strategies for designing helical peptidomimetics may feature stapling and *N*- and *C*-capping.^9^ Many stapling methods have been investigated in depth,^10,11^ and studies on *N*-cap mimics are common.^9,12,13^ However, as far as we are aware, only two papers^14,15^ mention synthetic *C*-cap mimics, and both comprise *monocyclic* rings. The first incorporated a 1,3-xylene linker between residue *C*3 and *C″* at the helix *C*-terminus with moderate helix-inducing effect, and the other is a dual-capped peptide (Figure 1e). The latter can only contain 9 helical residues, which limits its practical application in mimicking longer helices. Synthetic *C*-caps with high helix-inducing effects and free-to-extend *N*-terminus are required to develop this field. This paper describes a convenient and generalizable approach to smaller, inherently more rigid *bicyclic C*-capped helical mimics. It evolved from bioinformatic data collected on the most common *C*-cap motifs in proteins: Schellman loops (Figure 1).^16−18^

## Results and Discussion

**Figure 2:**
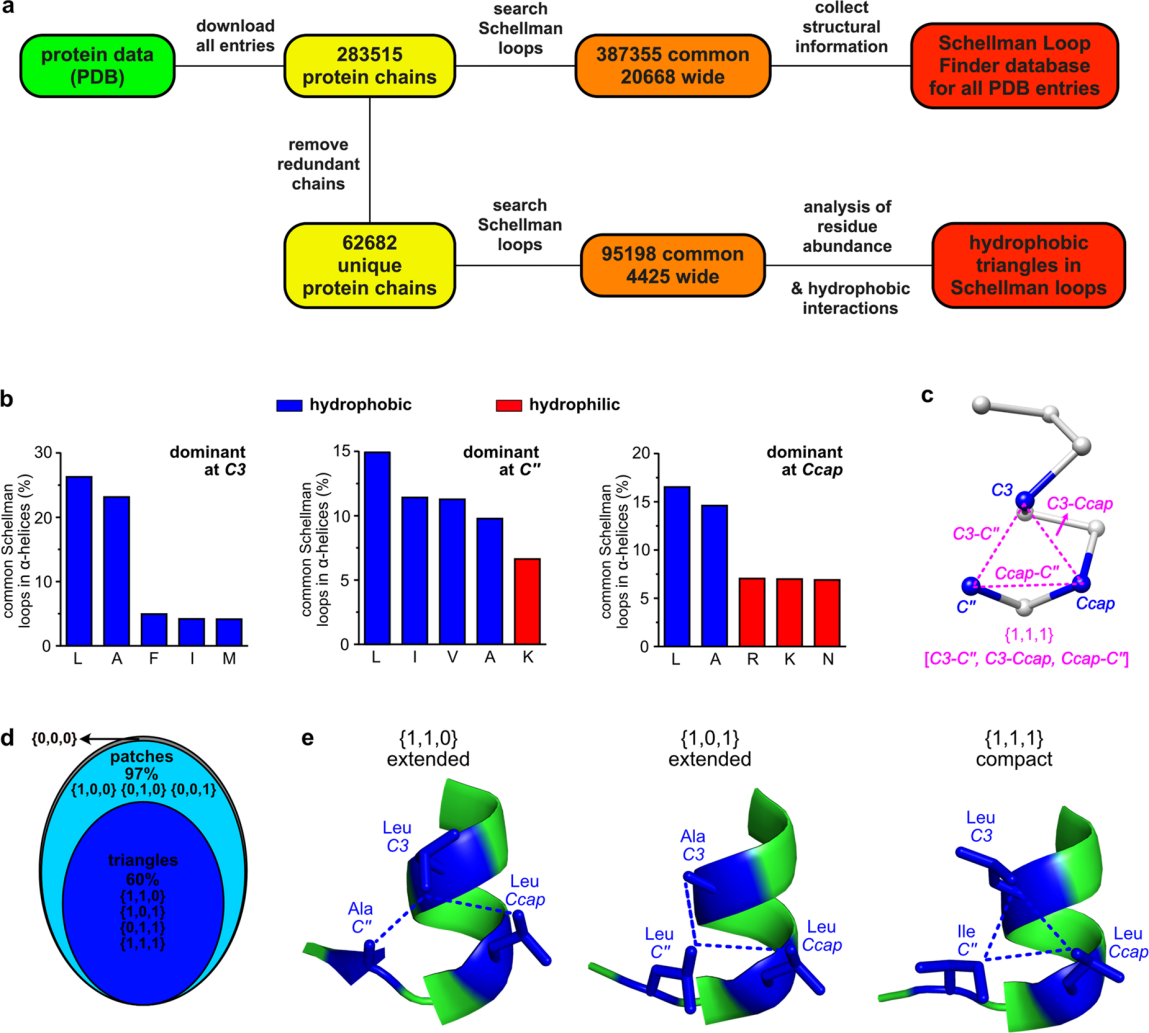
(a) Data from Schellman Loop Finder collected Nov 2020. (b) Prevalent residues at *C*3, *C″*, and *Ccap* in common Schellman loops (this paper uses “*C*-cap” to represent the whole motif and “*Ccap*” for the capping residue). (c) Three close contacts to form a {1,1,1} *hydrophobic triangle*. (d) Proportions of helical, common Schellman loops with hydrophobic *C*3 and *C″* that have no, one (*patches*), or twο or three (*triangles*) hydrophobic interactions. (e) Illustrative helical common Schellman loops in proteins with *extended* (two close contacts, {1,1,0} from PDB 4WJ3, {1,0,1} from PDB 4WJI) or *compact* (three) hydrophobic interactions (from PDB 5DLB), also illustrating the prevalent amino acids at each position. *Extended triangles* of the form {0,1,1} are rare (<0.5%; not shown).

Figure 2a outlines the bioinformatic workflow for this project. A custom script, Schellman Loop Finder, was developed by us. Using that, common and wide Schellman loops in proteins were searched and analyzed following a workflow with two branches. The first (Figure 2a, top) establishes a complete Schellman loop database which enables searching the motifs for any particular PDB entry. Beneath that, the other branch has duplicate chains removed^21^ to enable counting of *nonredundant Schellman loops* for statistical analyses. Key data on common forms (>95% of the total, Figure 1a) are presented here, and corresponding data on wide forms (Figure 1b) and detailed analyses of both types are described in Section C in the Supporting Information.

Figure 2b highlights most popular residues at *C*3, *C″*, and *Ccap* in helical common Schellman loops. The dominance of hydrophobic residues at *C*3 and *C″* was predictable because these tend to pack into *hydrophobic patches*.^22,23^ Preference of hydrophobic residues at *Ccap* was observed in an early study on a small data set (160 PDB entries containing 431 helices) without detailed analyses of how they might pack,^24^ and as far as we are aware, no studies have followed up on this point. As a result, a deeper study using our complete set of unique Schellman loops was facilitated to a better understanding of the packing of hydrophobic side chains at *C*3, *C″*, and *Ccap* in common Schellman loops.

A filtering script to identify potential hydrophobic contacts was made in-house. It requires that the nearest carbon atoms between two side chains (*C*3, *Ccap*, or *C″*) be less than 4.5 Å to ensure hydrophobic interactions. “Patches” were previously defined by one close interaction: *C*3–*C″*. However, our analyses have used a broader definition to include *C*3–*Ccap* and *C″*–*Ccap* because these are also interactions between two side chains. Data interpretation was facilitated by denoting the three possible interactions, *C*3–*C″*, *C*3–*Ccap*, and *Ccap*–*C″*, in binary nomenclature {X,X,X}. Thus, there are eight possibilities from {0,0,0} to {1,1,1} where 1 refers to potential interactions and 0 means none. A score of 1 in any position defines a patch. *Hydrophobic triangles*, a term proposed by us, are for those Schellman loops with at least two patches among *C*3, *Ccap*, and *C″*, because in such cases all three residues participate in the triangulated hydrophobic clusters. Figure 2c illustrates {1,1,1}: a typical hydrophobic triangle with all three possible hydrophobic interactions. Figure S7a shows all eight possible combinations, and Figure S7b gives a detailed breakdown of their relative proportions.

Of all Schellman loops with hydrophobic *C*3 and *C″* (38935), Figure 2d shows (i) that only 3% had no interactions; thus, (ii) 97% had at least a patch, and (iii) 60% had a triangle. Therefore, *hydrophobic triangles comprise the majority of the data set*. Figure 2e illustrates hydrophobic triangles can be *extended* where the three residues are inclined to be stacked, and within this group there are three possibilities: {1,0,1} (91% among all *hydrophobic triangles*; most frequently Ala,Leu,Leu, and Leu,Leu,Leu slightly less), {1,1,0} (2.9%; Leu,Leu,Ala) or, conceivably, {0,1,1} (not shown, <0.5%). Hydrophobic triangles can also be *compact* {1,1,1} (5.4%; most frequently Leu,Leu,Ile, and Leu,Leu,Leu slightly less). Leucine prevails over all other hydrophobic residues in these packing arrangements vaguely reminiscent of leucine zippers.^25^ Hydrophobic triangles stabilize Schellman loops by insulating the inner backbone conformations and intramolecular *H*-bonds important for helix-induction.

Conformations of peptide fragments isolated from their native protein environments can be different to those in the parent protein, especially if the fragment contains a hydrophobic motif which could pack against similar regions when buried inside the protein. To be sure that is applicable in this particular case, we examined hydrophobic triangles from our data set above, and found that 87% were fully buried and only 2% were completely at the solvent accessible surface of the parent proteins.

It follows from the analysis above that triangles in isolated model peptides would be exposed to solvent, and may not adopt the desired conformation. Nevertheless, we made short peptides with hydrophobic triangles to test if these motifs could measurably enhance helicities for isolated helical fragments in solution. Specifically, we prepared ones having the most abundant residue combinations in *compact* triangles (Figure S8): Ac-WAAAKAAAAKA**X**AA**X**G**X**-NH_2_ (**X**’s denote these key hydrophobic residues). Peptides using Leu,Leu,Leu or Leu,Leu,Ile as triangles presented enhanced helicities versus a control peptide with no Schellman loops or triangles, but these effects were small as anticipated (Figure S15). Thus, hydrophobic triangles could show a small but measurable impact on linear peptide fragments. This observation motivated us to consider more closely how hydrophobic triangles might be mimicked in *constrained* helical mimics, where the conformational restrictions might overcome the unfavorable hydrophilic–hydrophobic solvent interactions. Pursuit of that strategy led to the design of the mimics we call “BSMs” as described below.

**Figure 3:**
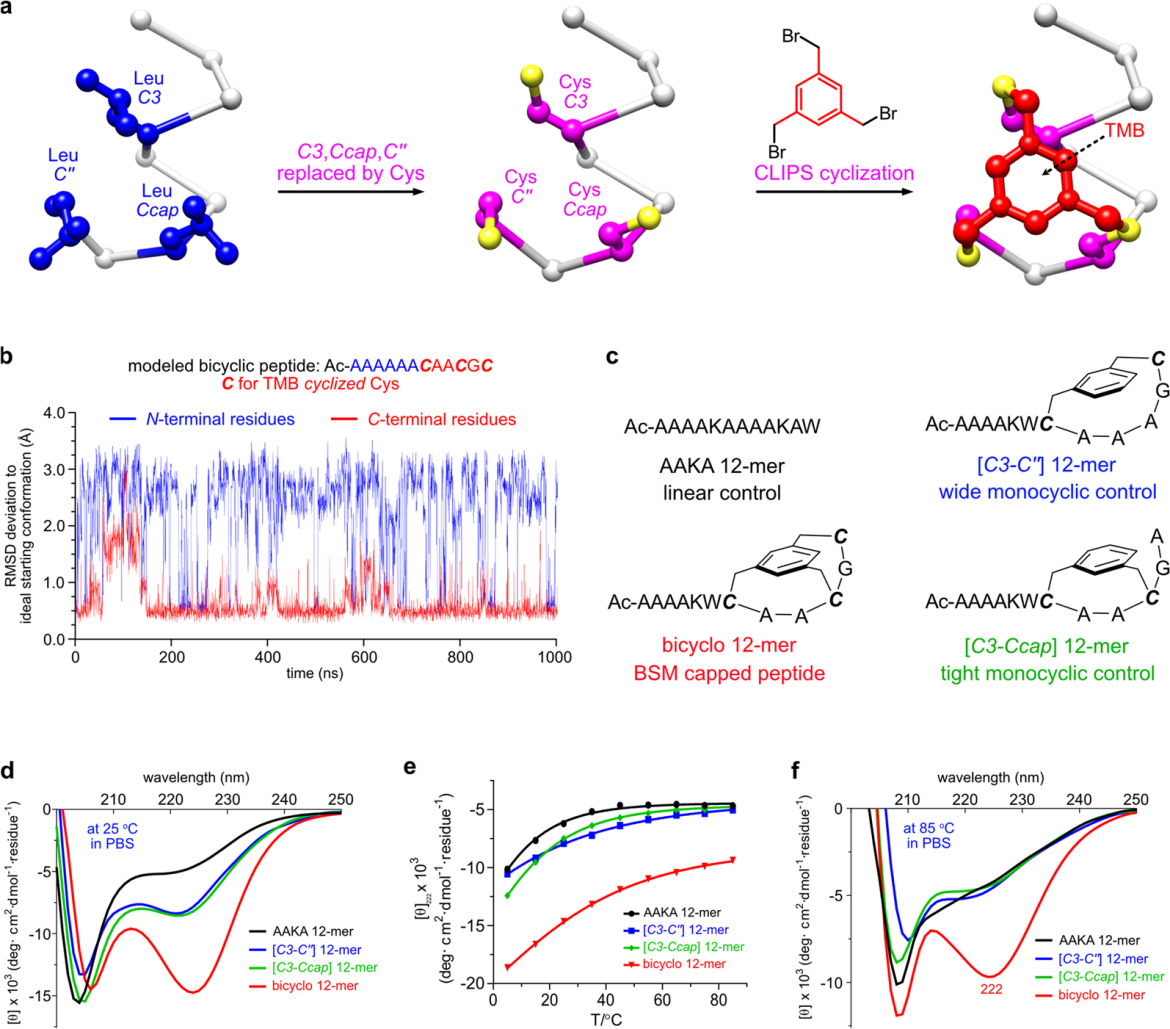
(a) Hypothesis: three hydrophobic triangle residues could be replaced by Cys and then cyclized with a TBMB to form bicyclic Schellman loop mimics. (b) Deviation of non-*H* backbone atoms in the 12-mer model peptide from an ideal starting conformation coordinates comprising an α-helix and a Schellman *C*-cap with the BSM modification in MD at 300 K. (c) Cartoon illustrating structures of the three controls, and the featured bicyclo 12-mer. (d) CD spectra of the linear (black), wide monocyclic (blue), and tight monocyclic (green) controls and bicyclo 12-mer (red) in PBS buffer at 25 °C. (e) Variation of molar ellipticities at 222 nm with temperature in PBS. (f) CD spectra of the peptide and peptidomimetics at 85 °C in PBS.

BSMs manifest the key innovation of this work: unification of the assembled hydrophobic triangles into a single trimethyl-benzenoid ring (Figure 3a). That mimic design has *C*3, *Ccap*, and *C″* replaced by three Cys and then capped with TBMB (1,3,5-tri{bromomethylene}benzene) to enforce the structural integrity of the *C-cap* conformations. Thus, we aspired to make bicyclic Schellman loop mimics (BSMs; these are, as one referee described them, “stapled Schellman loops”). TMB (1,3,5-tri{methylene}benzene) is an ideal framework for compact mimics because its three methylene groups are spatially equidistant like the corners of the near-equilateral hydrophobic triangle in these {1,1,1} situations.

A starting point was required to test the conformational rigidifying effect of BSMs via molecular dynamics (MD). Consequently, we virtually amalgamated coordinates of an ideal α-helix and a BSM *C*-cap to form the 12-mer peptidomimetic in Figure 3b (bold red ***C***s denote Cys capped by a TMB fragment). MD simulations were executed over 1 μs (explicit water box at 300 K); this relatively long simulation allows the mimic to reach stable conformations. Root mean square deviations (RMSD) of non-*H* backbone atoms relative to the “perfect” starting conformation were quantitated as a function of time to track unwinding of this ideal structure. In the experiment, the unconstrained *N*-terminus (blue in Figure 3b) continually flipped in and out of the ideal helical conformation, but the bicyclic *C*-cap integrity dominated throughout after fluctuation in the first 150 ns (red line). These observations on a simulated system suggested that the BSM framework is rigid and stable.

CLIPS (Chemical Linkage of Peptides onto Scaffolds) is emerging as the dominant click reaction in peptide chemistry,^26,27^ and it proved effective here. Crude tri-Cys peptide precursors could react with TBMB at 25 °C to give mainly (typically >90%) desired products within 15 min. Typical LCMS traces of crude products after cyclization are in Figure S13.

Three control compounds were made to compare with the featured BSM-capped peptide, i.e., “bicyclo 12-mer” (red; sequences in Figure 3c): (i) a linear control from natural amino acids (black); (ii) a monocyclic one representing Cys-to-Ala substitution of the second Cys in the featured compound, then cyclization with 1,3-di(bromomethylene)benzene, i.e., “wide monocyclic control” (blue); and, (iii) another monocyclic control representing Cys-to-Ala substitution of the third Cys then cyclization with the same electrophile, i.e., “tight monocyclic control” (green). These two monocyclic controls are mimics of *C*3–*C″* and *C*3–*Ccap* hydrophobic patches, respectively, and can be used to examine if the bicyclic triangle mimic is truly superior to monocyclic patch mimics. The fourth conceivable control (a monocycle encompassing the *C*-terminal **C**G**C**) was not considered because those three residues in the parent Schellman loop are outside the helical region and would not be expected to exhibit helicity.

Circular dichroism (CD) spectra of the BSM-containing bicyclo 12-mer and the three controls were recorded to evaluate their helicities. At 25 °C, the bicyclo 12-mer (red line) was significantly more helical than the others (Figure 3d). This was confirmed by two helical indicators: percent helicity and ellipticity ratio^12,28−30^ (Table 1). Both monocyclic controls had about the same helicities: more than the linear, but less than the bicyclic. Differences between bicyclo 12-mer and the three controls were even more conspicuous in the variable temperature studies (VT-CD; Figure 3e); the featured compound clearly had a more robust helicity. At high temperature, molecules are usually less ordered. This is the case for three controls: their 222 nm ellipticities converged to around −5000 (Figure 3e), and corresponding CD spectra at 85 °C resembled random coils (Figure 3f). However, bicyclo 12-mer had twice the 222 nm ellipticities than controls as temperature rose, and its CD spectrum at 85 °C still had significant α-helix characteristics. This shows that the helix-inducing BSM *C*-cap has surprisingly high thermal stability. Figures S16 and S17 and the Supporting Information describe parallel studies featuring experiments in 20% TFE/PBS, and 17-mer analogues. Similar trends were observed throughout.

**Table 1:** Ellipticity Ratios 222/208 nm and Percent Helicity for Four 12-mer Peptides at 25 °C in PBS Buffer

label	[θ]_222_/[θ]_208_	% helicity
AAKA 12-mer	0.31	17
[*C*3–*C″*] 12-mer	0.55	34
[*C*3–*Ccap*] 12-mer	0.62	33
bicyclo 12-mer	1.00	59

**Figure 4:**
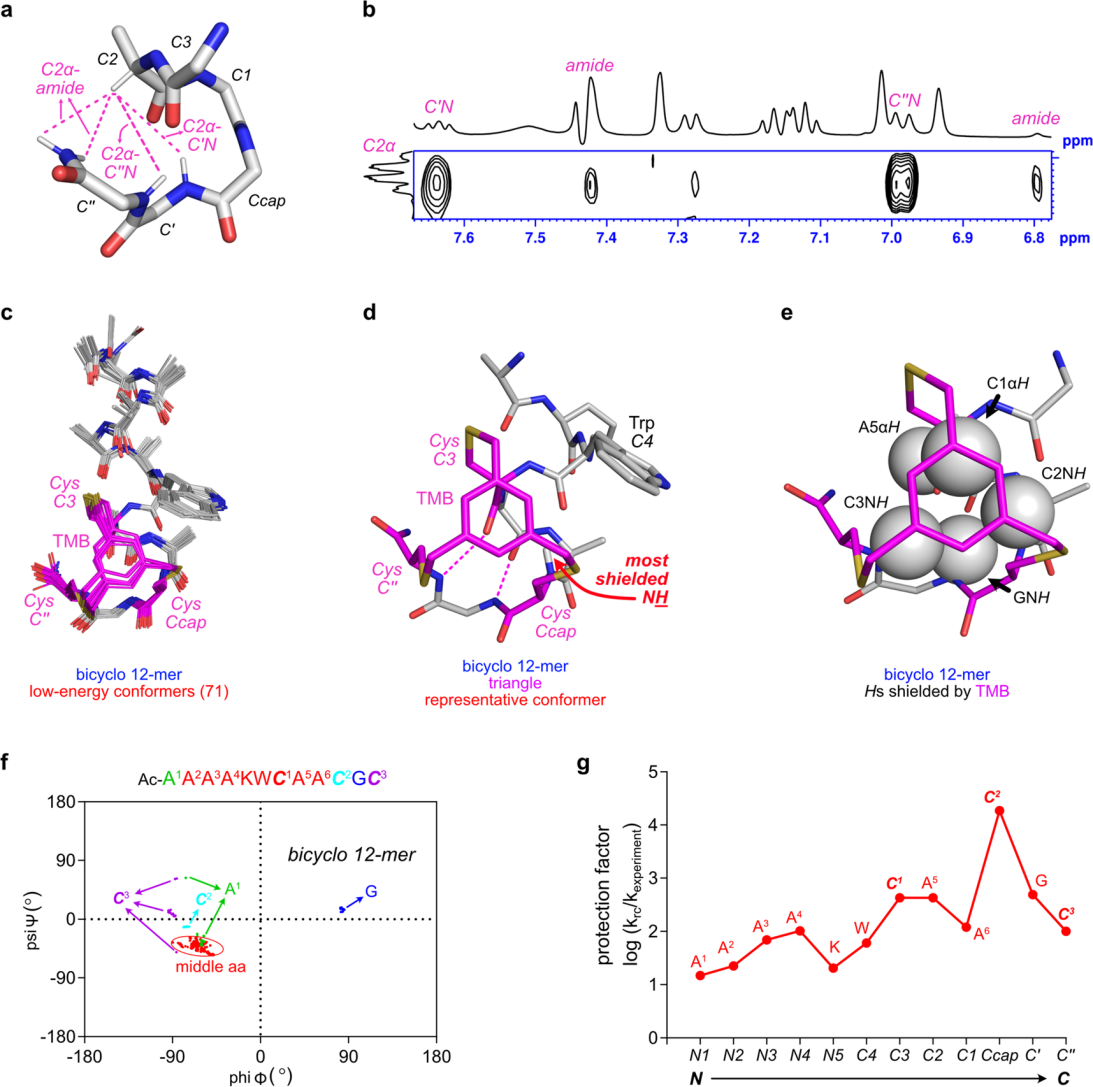
(a) Characteristic NOE cross peaks expected for a typical common Schellman loop, corresponding to (b) ones observed experimentally for bicyclo 12-mer. (c) All 71 low-energy conformers generated by MD simulations, (d) lowest-energy conformer which is also the representative of that set, and (e) diagram of that conformation showing five *H*-atoms that are likely to be shielded by the TMB group. (f) Ramachandran plot for 71 low-energy conformers of bicyclo 12-mer. (g) Protection factors^31^ for the *N*H in bicyclo 12-mer, where the red letters above the data points denote residues in the sequence, while the *x*-axis shows their positions in the helix-cap motif.

NMR studies gave further evidence supporting the proposed dominant *C*-capped helical conformation of bicyclo 12-mer. Four characteristic contacts anticipated for a *C*-carboxyamidated Schellman loop were all observed in the ROESY spectrum of the bicyclo 12-mer (Figure 4a,b). Relatively low NOE intensities between *C2α**H* and *C*-terminal *amide**H* are probably due to fast *H*/*D* and *H*/*H* exchanges between the terminal amide and nearby solvent molecules. Simulations using those and other NMR constraints gave very similar low-energy conformations (within 3 kcal/mol to the lowest one) except for the acetyl groups at the *N*-terminus (Figure 4c, Figure S27). Figure 4d shows the lowest-energy conformer with two *H*-bonds characteristic of common Schellman loops. Five hydrogens (2 *Cα**H* and 3 *N**H*) with smaller chemical shifts than their colleagues imply that they are probably shielded by TMB, which is consistent in the simulated conformers: these five hydrogens are behind TMB and therefore affected by its shielding effect (Figure 4e). This indicates that the TMB group forms a solid waterproof wall above the backbone of the Schellman loop (Figure S26).

A Ramachandran plot (Figure 4f) features all bicyclo 12-mer residues of the 71 low-energy conformers shown in Figure 4c. Residue ***C***^**2**^ in the BSM corresponds to *Ccap*. That residue and others downstream of it toward the *C*-terminus are expected to break from helicity, and the result matches well: ***C***^2^, G, and ***C***^3^ indeed do *not* have helical φ,ψ dihedral angles, whereas all the ones anticipated to be helical do. Even AcA^1^ at the *N*-terminus, where most deviation would be expected, is mostly helical. Finally, dihedrals of G at *C′* accurately fall into the region of left-handed α-helices, as often observed in Schellman loops.

*H*/*D* exchange experiments were conducted to measure protection of each carbonyl-amide *H*-bond. Predicted rates of exchange for the amide *H*’s in random coils were divided by the measured values to give protection factors. A convex curve is usually anticipated in peptides since *H*-bonds at the termini are destabilized. However, in the bicyclo 12-mer, there is no obvious fading at the *C*-terminus. This again marks the solid waterproof wall built by the TMB fragment. Incidentally, the *NH* of ***C***2 is particularly protected (Figure 4g); we think this is probably because of cooperation of TMB and the proximal *C*4 Trp (Figure 4d).

**Figure 5:**
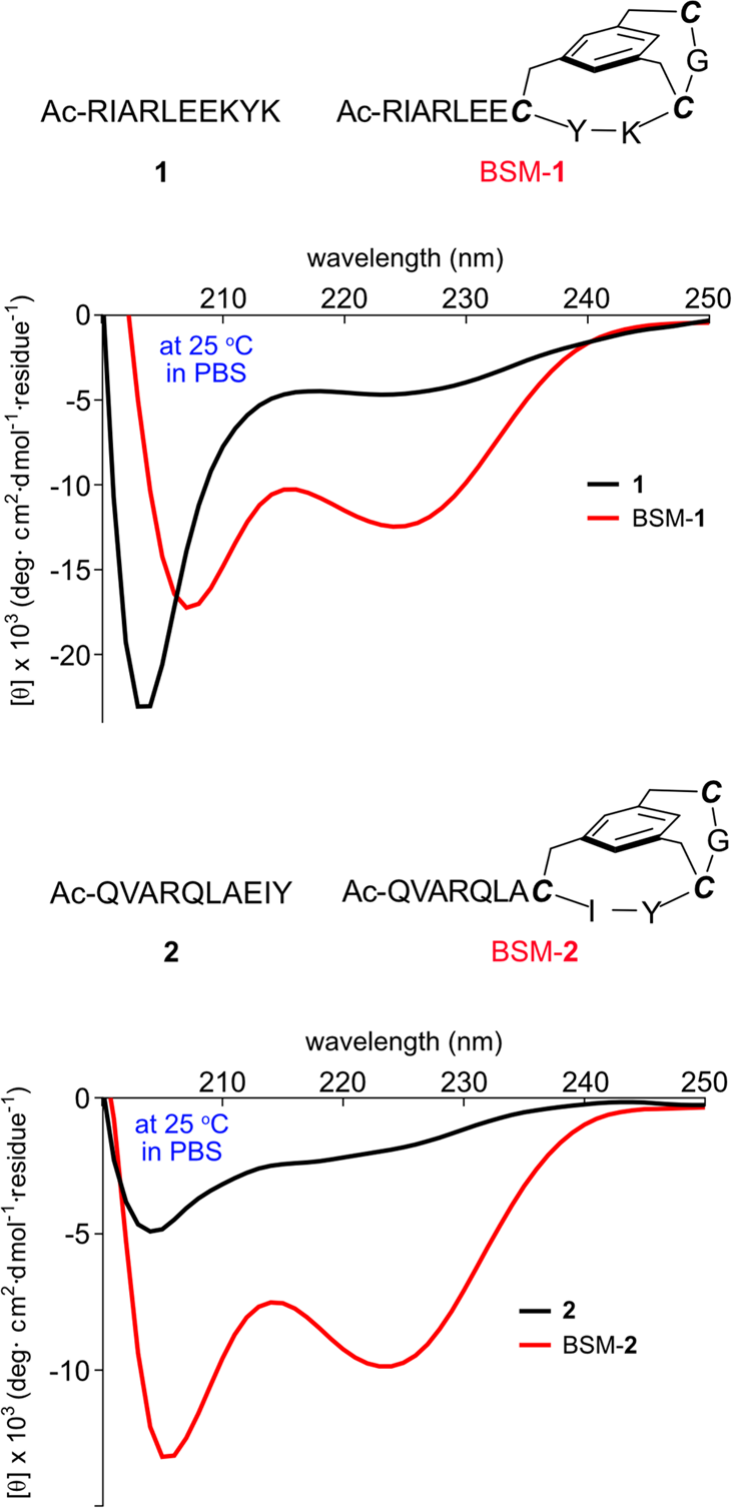
CD spectra compared for two fragments of peptides which occur in proteins (black lines) with the corresponding BSM mimics in PBS buffer at 25 °C.

Bicyclo 12-mer and the 17-mer described in the SI are Ala rich peptides which are usually biased toward helices. To test generality, peptides **1** and **2** were selected because they are not Ala rich, and have been used by others to test enforced helicity.^12^ Sequences **1** and **2** correspond to helical fragments in proteins which are not helical as isolated peptides in aqueous solution; they are hydrophilic and hydrophobic, respectively, and hence provide different types of comparisons. Consequently, we prepared these and their BSM mimics BSM-**1** and BSM-**2** (Figure 5). To make BSM mimics of any sequence, two changes are required. One is that the residue at *C*3 will be mutated by Cys. The other is that three residues, Cys-Gly-Cys, will be added after the last expected helical residue in the sequence. These three residues correspond to *Ccap*, *C′*, and *C″*, and are not helical, so they cannot mutate expected helical residues unless a mimic of truncated helix is desired. Since all residues in **1** and **2** were expected to be helical, three more residues were extended at their *C*-termini to form BSM mimics. Helicities of the peptides and peptidomimetics were compared on a per-residue basis. Figure 5 shows that neither of the control peptides have CD spectra resembling α-helices, but BSM-**1** and BSM-**2** do; the percent helicity and pertinent ellipticity ratios are given in Table 2. This proves that the BSM capping method can be applied in more general situations.

**Table 2:** Ellipticity Ratios 222/208 nm and Percent Helicity for Biological Peptides and BSM Analogues at 25 °C in PBS Buffer

label	[θ]_222_/[θ]_208_	% helicity
**1**	0.19	17
BSM-**1**	0.72	48
**2**	0.40	8
BSM-**2**	0.76	38

## Conclusion

In conclusion, *hydrophobic triangles comprise the majority of the data set of helical Schellman loops* (Figure 2d). This significant observation led us to the biomimetic approach in Figure 3 which could not have been conceived otherwise. BSMs are conveniently accessible via inherently efficient CLIPS reactions. Their unique *bicyclic* structure makes them more constrained and, presumably, more helix-inducing than the *C*-cap mimics reported to date which are *mono*cycles. In fact, the cap motif of [*C3–C″*] 12-mer, one of our monocyclic controls which is less helical, is similar to a reported *C*-cap system.^15^ BSMs support helical conformations well, even in short peptides and at elevated temperatures (Figure 3). Our bioinformatics also showed that Schellman loops seldom contain hot spot residues (Figure S12 and the Supporting Information). Consequently, substitutions of three Cys residues to form BSMs would not deter from their use in interface mimicry, because residues corresponding to hot spots are usually incorporated outside the *C-cap*. Besides, BSM can also be applied on helical fragments without Schellman loops (Figure 5). This requires the extension of three amino acids CGC at the end of the last helical residue, to form BSM mimics. Significant improvement of helicity for short biologically relevant peptides confirmed the feasibility of the new *C*-capping method in peptides to give mimics with different physiochemical properties.

## Supporting Information Available

Details of data mining and hot loop search procedure; statistical data from generated Schellman loop database; results of hot loop analysis; synthesis procedure; details of CD experiments with procedure, raw data, and analyses; MD analysis; details of NMR experiments with procedures, raw spectra, and constraints; and characterization of peptides (PDF)
Schellman loop Finder Database (ZIP)
Nonredundant Schellman loops (ZIP)
α-Helical wide, common, and π-helical wide Schellman loop hydrophobic patterns (ZIP)
Hot Schellman loops (ZIP)
NMR solution ensembles of bicyclo 12-mer uploaded as a .pse file (ZIP)
