# Supplementary material for: Bicyclic Schellman Loop Mimics (BSMs): Rigid Synthetic C-Caps for Enforcing Peptide Helicity
Source: ACS Cent Sci. 2023 Feb 13;9(2):300–6. doi: 10.1021/acscentsci.2c01265 (PMC9951308; doi:10.1021/acscentsci.2c01265)
Supplement: Supplementary file 1 — oc2c01265_si_001.pdf [file oc2c01265_si_001.pdf]

Bicyclic Schellman Loop Mimics (BSMs): Rigid Synthetic *C*-Caps For Enforcing Peptide Helicity

Tianxiong Mi, Duyen Nguyen, Kevin Burgess*

*Department of Chemistry, Texas A & M University, Box 30012, College Station, TX 77842, USA

E-mail: burgess@tamu.edu

Table of Contents

A. Abbreviations **3**
B. Datamining to Find Schellman Loops in Protein Databank (PDB) **4**
Schellman Loop Finder Database 4
Hot Schellman Loop Dataset 5
Nonredundant Schellman Loop Dataset for Statistical Analysis 5
C. Statistical Analysis on Nonredundant Schellman Loop Dataset **6**
Common and Wide Schellman Loops 6
Detailed Distribution of Common and Wide Schellman Loops in Different Secondary Structure 7
Dihedral Angles of Common and Wide Schellman Loops 9
Residue Abundancy from *C3* to *C''* in Common Schellman Loops 10
Residue Abundancy from *C4* to *C''* in Wide Schellman Loops 12
Search of Hydrophobic Patch and Triangle in *C-cap* Common Schellman Loops 14
Residues Preference at (C3, Ccap, C'') in Different Hydrophobic Patterns 15
How many hydrophobic triangles are at surfaces of parent proteins (chains)? 16
Search of Hydrophobic Patch and Triangle In *C-cap* Wide Schellman Loops 17
Wide Schellman Loops at α-helix C-termini 17
Wide Schellman Loops at π-helix C-termini 18
D. Hot Segment Analysis on Interface Schellman Loop Dataset **19**
Hot Interface Schellman Loops 19
E. Syntheses of Peptides **20**
Linear Peptides 20
Bicyclic Capped Peptides 21
F. Circular Dichroism (CD) Experiments **23**
Calculation of percent helicity 23
CD spectra of synthesized 17-mer linear peptides in PBS buffer 24
CD spectra of synthesized 17-mer bicyclic peptides in PBS buffer 25
CD spectra of synthesized 12-mer peptides in 20% TFE/PBS buffer 26
Variable Temperature CD Experiment 27
G. MD on Modeled Bicyclic *C*-capped 12mer **28**
H. NMR Experiments **29**
1D and 2D NMR Spectroscopy 29
Bicyclo 12-mer 29
NMR Spectra 29
Peak Assignment 34
Distance and Dihedral Constraints 34
NOE Summary 40
Ramachandran Plot of (θ, ψ) Angles 41
The Impacts of Shielding Effect from TMB to Nearby Hs 42
Solution Structural Ensembles 43
Amide H-D Exchange Study 44
Bicyclo 12-mer H-D Exchange Study 44
Fitting Curves And Summary 45
I. Characterization of Purified Peptides **46**
J. References **51**

A. Abbreviations

TLC: thin layer chromatography

prepHPLC: preparation high performance liquid chromatography

MeCN: acetonitrile

analyHPLC: analytical high performance liquid chromatography

ESI-MS: electrospray ionization mass spectrometry

Fmoc: fluorenylmethoxycarbonyl

DIPEA: N,N-diisopropylethylamine

DMF: dimethylformamide

Oxyma: ethyl cyanohydroxyiminoacetate

DIC: N,N'-diisopropylcarbodiimide

PyBOP: benzotriazol-1-yloxytripyrrolidinophosphonium hexafluorophosphate

HOBt: hydroxybenzotriazole

NMM: N-methylmorpholine

TFA: trifluoroacetic acid

TFE: 2,2,2-trifluoroethanol

TIPS: triisopropylsilane

DMSO: dimethyl sulfoxide

PBS: phosphate-buffered saline

MCMM: Monte Carlo Multiple Minimum

TBMB: 1,3,5-tris(bromomethyl)benzene

TMB: 1,3,5-trimethylbenzene

B. Datamining to Find Schellman Loops in Protein Databank (PDB)

Schellman Loop Finder Database

(i) All entries from PDB were downloaded to a local computer. Till Nov. 2020, there were 1.7×10^5 entries.

(ii) 6.0×10^5 chains were found in these entries. A filter was implemented to remove chains of nucleic acids. Besides, for each entry, only one unsymmetrical unit was kept for further calculation, thus 2.8×10^5 chains remained. By doing this, all entries were kept while the computational time can be significantly reduced.

(iii) For chains in each entry, Schellman loops were searched based on following criteria

- a) Common Schellman loops:
  - i. H-bond pattern:
    1. $d(O_i – N_{i+5}) < 3.5Å$
    2. $d(O_{i+1} – N_{i+4}) < 3.5Å$
  - ii. Dihedral constraints:
    1. Right-handed Schellman loops:
       - a) Φ(phi) (i+1) < 0
       - b) Φ(phi) (i+2) < 0
       - c) Φ(phi) (i+3) < 0
       - d) Φ(phi) (i+4) > 0
    2. Left-handed Schellman loops (rare):
       - a) Opposite to above
- b) Wide Schellman loops:
  - i. H-bond pattern:
    1. $d(O_i – N_{i+6}) < 3.5Å$
    2. $d(O_{i+1} – N_{i+5}) < 3.5Å$

(iv) 3.9×10^5 common Schellman loops and 2.1×10^4 wide Schellman loops were found based on the criteria above for 2.8×10^5 chains.

(v) Structural information including sequence, dihedral angles and interface properties (at interface or not) were collected for these Schellman loops.

Hot Schellman Loop Dataset

Hot spot analysis were conducted using solvent accessible surface area (SASA) algorithm[1]. Only Schellman loop segments were studied in this dataset. Interface Schellman loops were collected and the following parameters were calculated:

a) *ΔSASA(X)*: The change of solvent accessible surface area for X before and after binding the receptor. The parameter can be regarded as the area of X that binds to the receptor. Each residue in Schellman loop was assessed for ***hot residue*** by following criteria:
   i. *ΔSASA(residue).*
      For a hot spot, we required the value to be higher than 50 Å^2.
   ii. $\frac{\Delta SASA(residue)}{SASA(residue\ in\ free\ ligand)}$.
      For a hot spot, we required the ratio to be higher than 90%, so that most of the solvent accessible area of the residue would be buried by the receptor.

b) $\frac{\Delta SASA(Schellman\ loop)}{\Delta SASA(chain)}$
   The parameter compares the binding area of Schellman loop (on ligand protein) with that of the ligand. A high percentage here means the binding of ligand and receptor is dominated by the Schellman loop.

c) $\frac{\Delta SASA\ (Schellman\ loop)}{number\ of\ residues}$
   The parameter (Å^2) calculates the average binding area for each residue of the Schellman loop. A high value here means the Schellman loop contributes a big binding area to the receptor. It can be a complement to b) because there were cases with high percentages but very little binding areas.

d) All interface Schellman loops were calculated to obtain parameters a), b) and c), and by considering them together it is possible to find cases matching the requirements. For example, by setting b) > 10% and c) > 50 Å^2, Schellman loops matching the requirements will be filtered.

Nonredundant Schellman Loop Dataset for Statistical Analysis

The dataset for statistical analysis includes Schellman loops only from nonredundant chains. Steps (i), (iii), (iv) and (v) are the same as above, and the only difference is on step (ii) in *Schellman Loop Finder Database*.

(ii) 6.0×10^5 chains were found in these entries. Clustering calculation based on sequence similarity for these chains was implemented using CD-HIT web server[2,3] with an identity cutoff = 0.95. It is assumed that chains with such high sequence similarity would have similar conformations and only need to be considered once. At last, 6.3×10^4 unique chains were obtained for further analysis.

All generated datasets can be found at the GitHub page:

C. Statistical Analysis on Nonredundant Schellman Loop Dataset

Common and Wide Schellman Loops

Grid lines in **Fig S1a** represent the percentage of motifs at the *C*-termini of α-helices in each class. Overall, more than 80% common Schellman loops are at the *C*-termini of α-helices. While in wide Schellman loops, 60% are at *C*-termini of α-helices and more than 1/4 are located at the *C*-termini of π-helices.

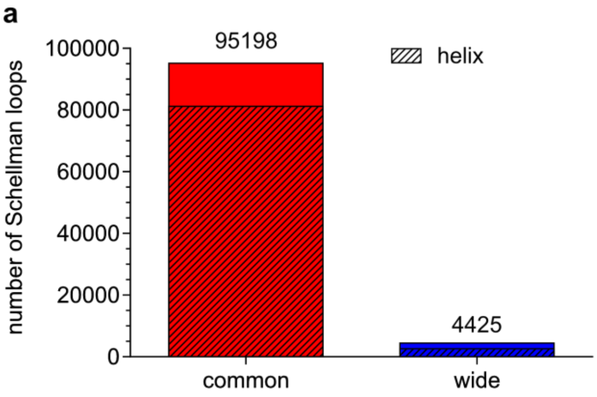

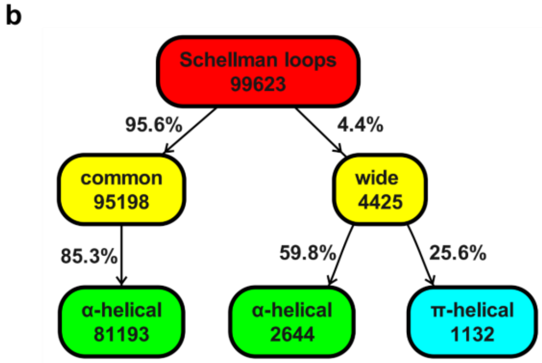

Fig S1 **a**. number of Schellman loops in different classes. **b**. number of helical motifs in common and wide Schellman loops.

Detailed Distribution of Common and Wide Schellman Loops in Different Secondary Structure

For each motif, *C3,C2,C1,Ccap,C',C''* (+ *C4* in wide type) were used in the secondary structure analyses with the help of *DSSP*[4] program. Those with same secondary structure combos were combined and divided total number of motifs in that class to obtain the fraction and make the graphics.

In common Schellman loops, majority (>85%) are at *C*-termini of α-helices, acting as *C*-caps. The others mainly show up at turns and strands, or at termini of narrower 3/10 helices.

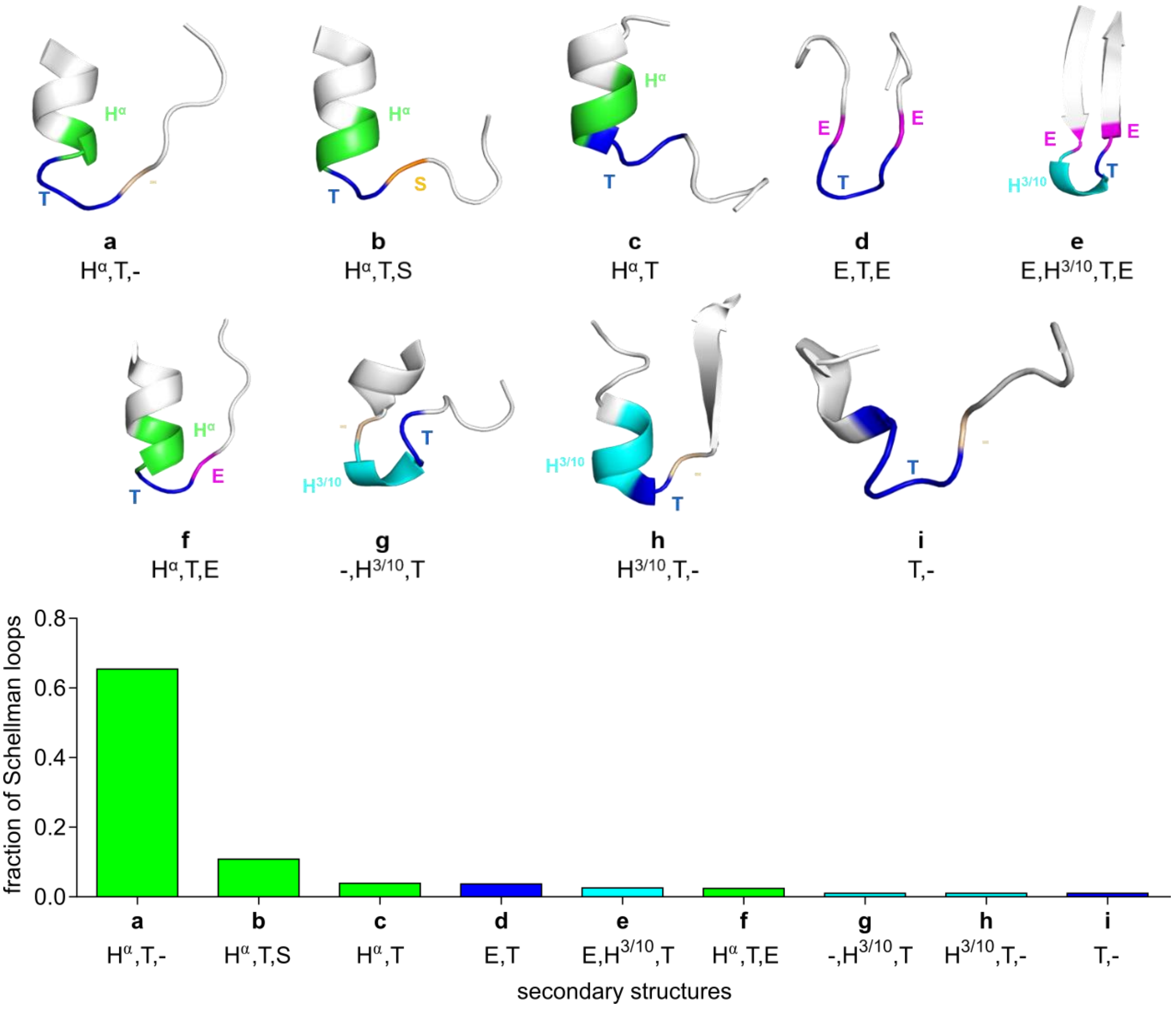

Fig S2 Top 9 secondary structures in common Schellman loops

In wide Schellman loops, majority (~85%) are at *C*-termini of helices, acting as *C*-caps. However, around 2/3 of these appear at the end of α-helices, while the other 1/3 at π-helices, perhaps due to their more expansive conformation.

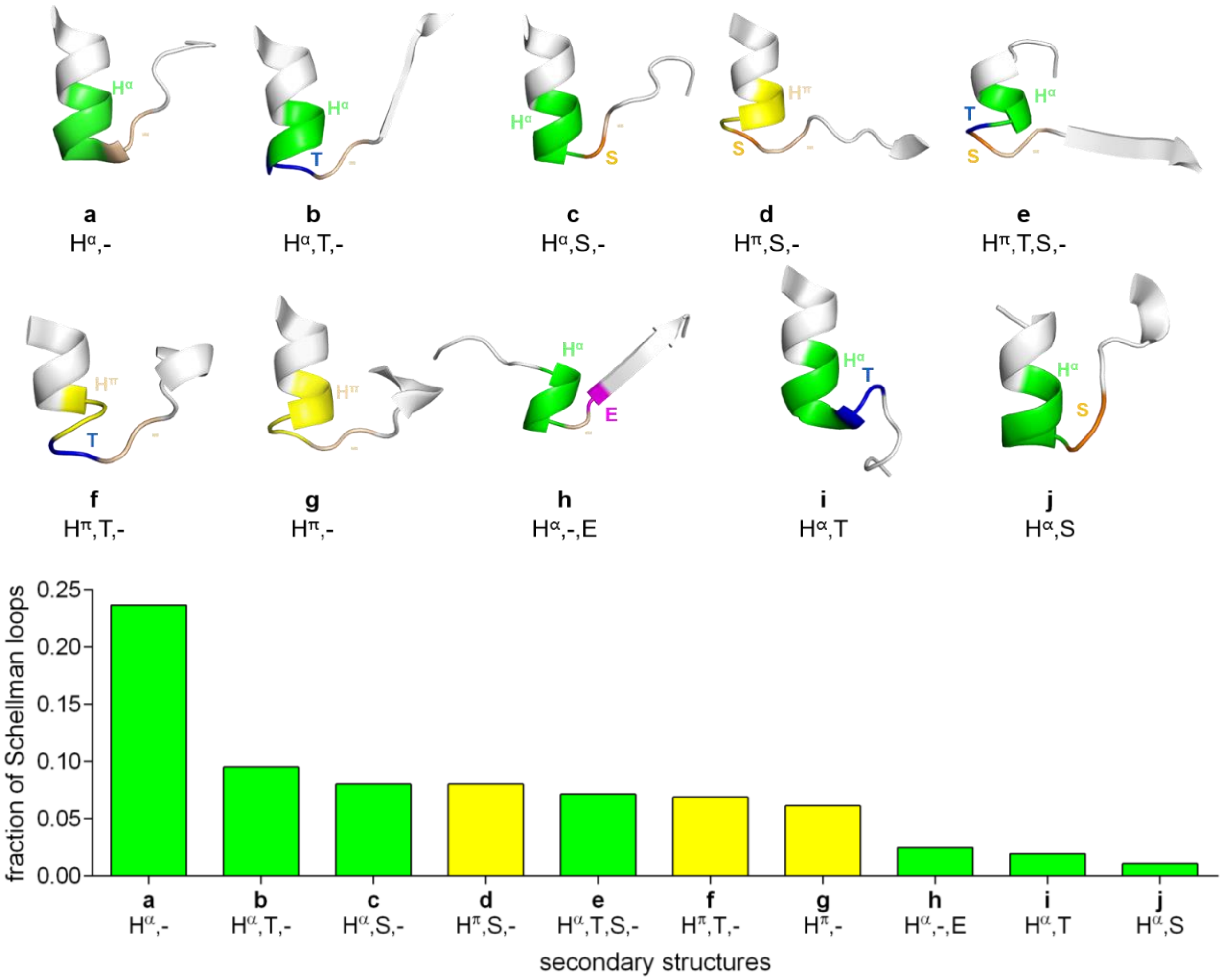

Fig S3 Top 10 secondary structures in wide Schellman loops

Dihedral Angles of Common and Wide Schellman Loops

Dihedral angles analyses were made to 81193 common and 2644 wide helical terminal Schellman loops. It indicated that they were helix-inducing motifs and helix usually started at *C1*. Comparing the two types, common Schellman loops have smaller deviations to standard α-helix (-60°, -40°) from *C1* to *C3*.

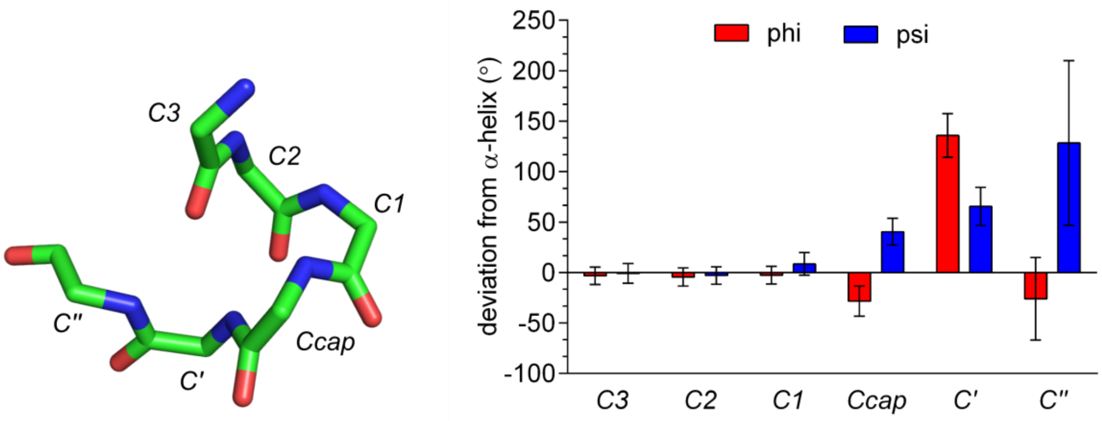

common Schellman loops at α-helical *C*-termini

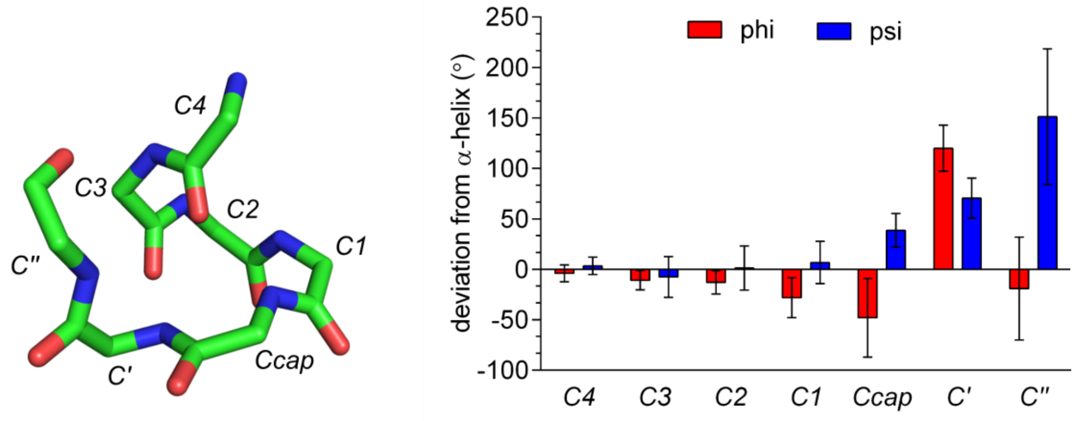

wide Schellman loops at α-helical *C*-termini

Fig S4 Dihedral angles {relative to (-60,-40°)} of helical common and wide Schellman loops.

Residue Abundancy from *C3* to *C''* in Common Schellman Loops

Common Schellman loops were collected and grouped into two classes based on their secondary structures: at α-helix terminus (81193) *vs* not at α-helix terminus (14005). Here residue abundance in these classes were computed to study residue preferences in different secondary structures. In each class, residues at the same position (*C3*, *C2*, *C1*, *Ccap*, *C'* and *C''*) (in non-helical Schellman loops, their corresponding labels are *i+3*, *i+2*, *i+1*, *i*, *i-1*, *i-2*) were collected, grouped and counted. The values divided total number of motifs in the class to obtain fractions. The top five residues at each position were presented and compared.

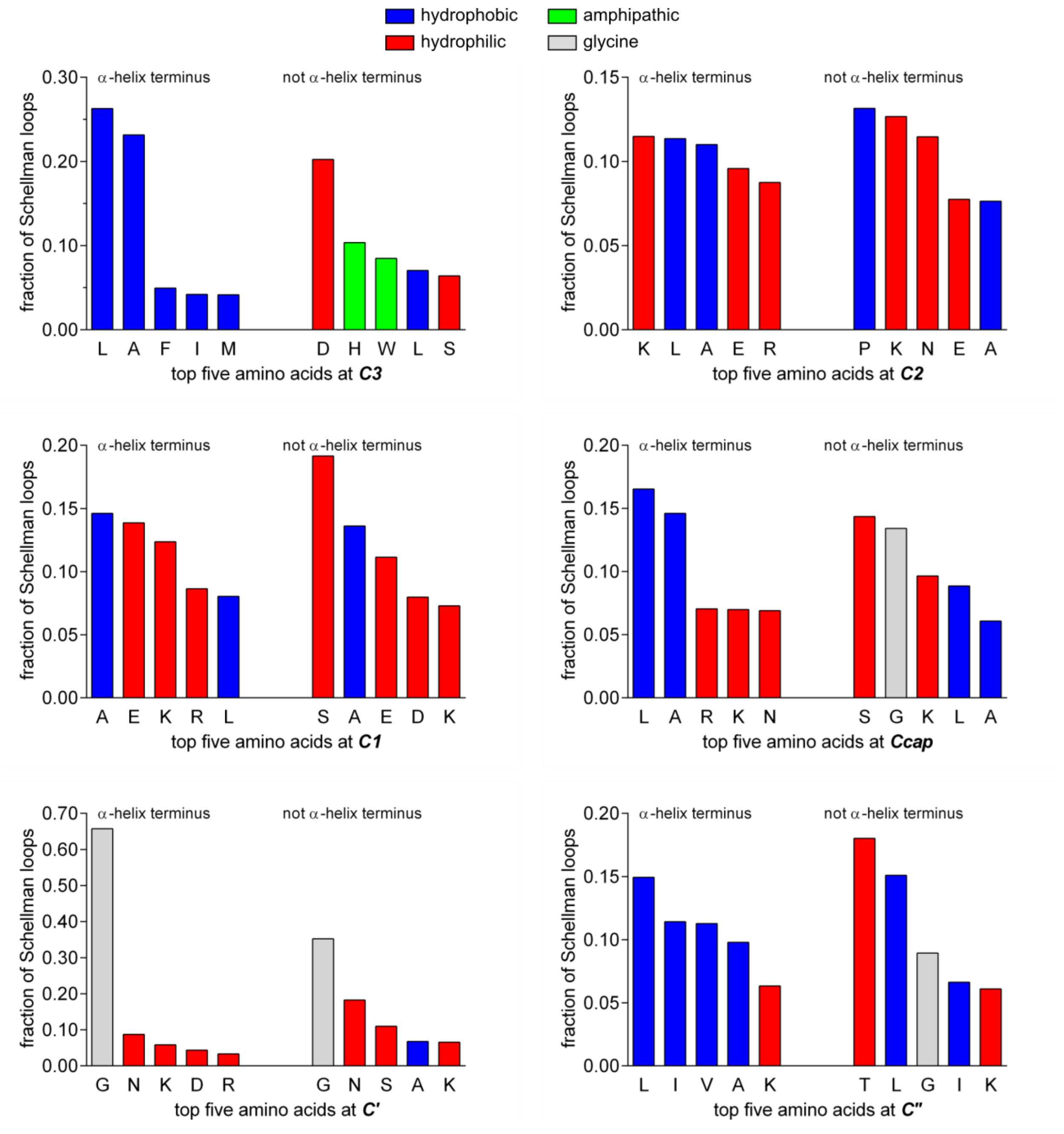

Fig S5 Top 5 most abundant residues from *C3* to *C''* in common Schellman loops.

Comparing residue abundance in two classes, there are a few significant difference:

(i) Hydrophobic residues are very abundant at *C3* and *C''* when Schellman loops are at α-helix termini, while much less so when not.

(ii) Gly are favored at *C'* in both classes, but much more preferrable when at α-helix termini, even more than 60%. This probably demonstrates the importance of using residues capable of left-handed conformations at *C'*.

(iii) Hydrophobic residues L and A are preferred at *Ccap* when at α-helix termini, but much less abundant when not. It indicates they might have some functions for Schellman loops at α-helix termini, for example, stabilizing the conformations somehow.

Residue Abundancy from *C4* to *C''* in Wide Schellman Loops

Wide Schellman loops were collected and grouped into three classes based on their secondary structures: at α-helix terminus (2644) *vs* at π-helix terminus (1132) *vs* not at helix terminus (649). Here residue abundance in these classes were computed to study residue preferences in different secondary structures. In each class, residues at the same position (*C4*, *C3*, *C2*, *C1*, *Ccap*, *C'* and *C''*) (in non-helical Schellman loops, their corresponding labels are *i+4*, *i+3*, *i+2*, *i+1*, *i*, *i-1*, *i-2*) were collected, grouped and counted. The values divided total number of motifs in the class to obtain fractions. The top five residues at each position were presented and compared.

Comparing residue abundance in the three classes, there are a few significant difference:

(i) Hydrophobic residues are very abundant at *C4* and *C''* when Schellman loops are at (α- and π-) helix termini, while much less so when not.

(ii) Hydrophobic (including some amphipathic) residues are abundant at *Ccap* when at helix termini, especially in π-helix termini, but much less abundant when not. It indicates they might have some functions for Schellman loops at helix termini, for example, stabilizing the conformations somehow.

(iii) Hydrophilic residues are very abundant at *C2* when at helix termini, especially in π-helix termini, but much less abundant when not. It might indicate *C2* is inclined to be solvent exposed for helical terminal wide Schellman loops.

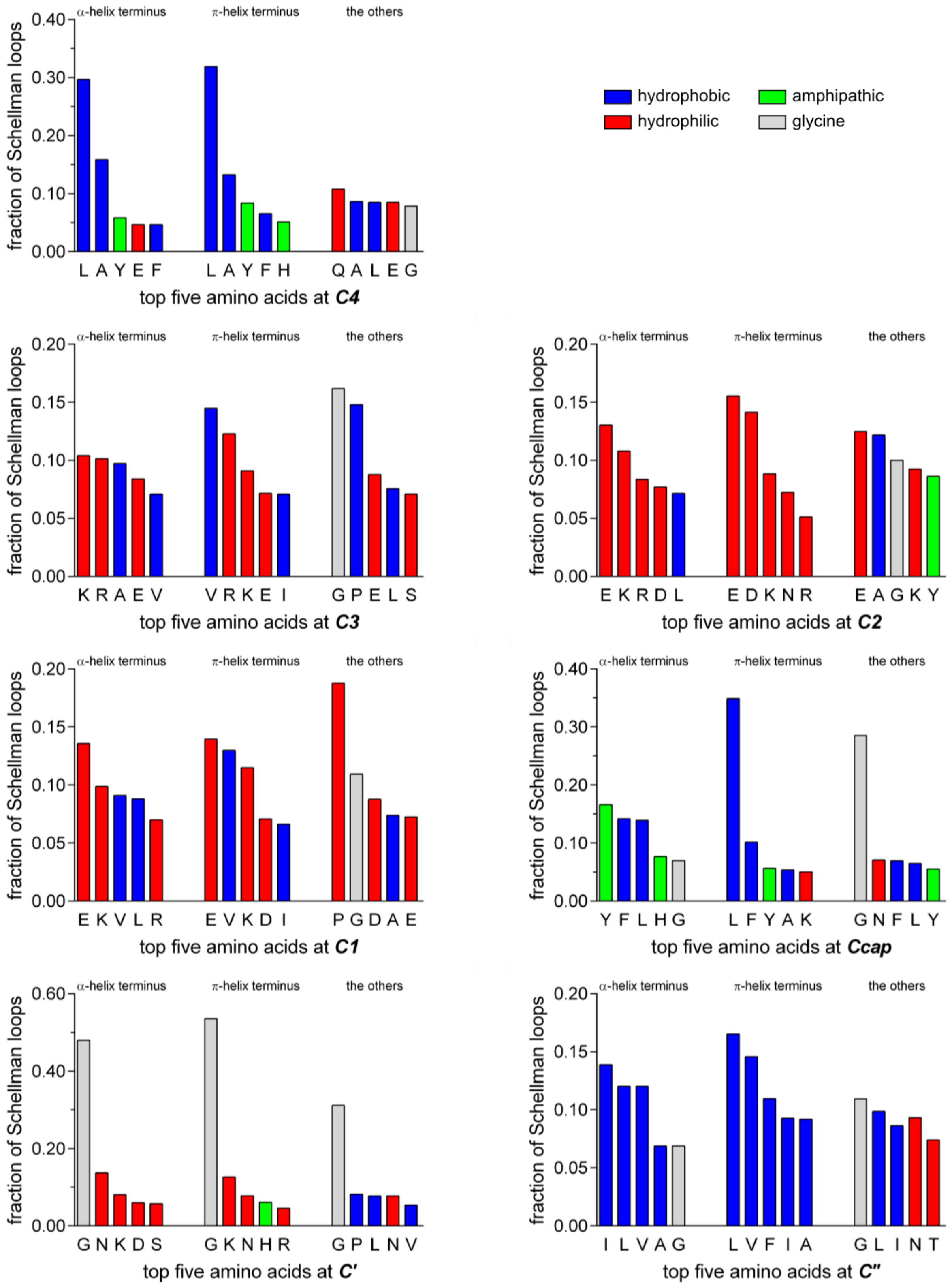

Fig S6 Top 5 most abundant residues from *C4* to *C''* in wide Schellman loops

Search of Hydrophobic Patch and Triangle in *C-cap* Common Schellman Loops

A script was designed to search if the hydrophobic interactions between sidechains of (*C3*, *C''*), (*C3*, *Ccap*) and (*Ccap*, *C''*) were possible to happen. The nearest **carbon** distance between sidechains of residue pairs was measured in the script, and if the distance was smaller than 4.5 Å, we assumed it was possible to have hydrophobic interactions between the two sidechains. A 3-element 1d array was used to describe the results, for example, if a motif was possible to have interactions among (*C3*, *C''*), (*C3*, *Ccap*) and (*Ccap*, *C''*), the result for it would be {1,1,1}.

Among 81193 common Schellman loops at α-helical *C*-cap, 38935 which had hydrophobic residues at *C3* and *C''* were used in this study. Eight different patterns were found, and numbers of them are shown in Fig S7.

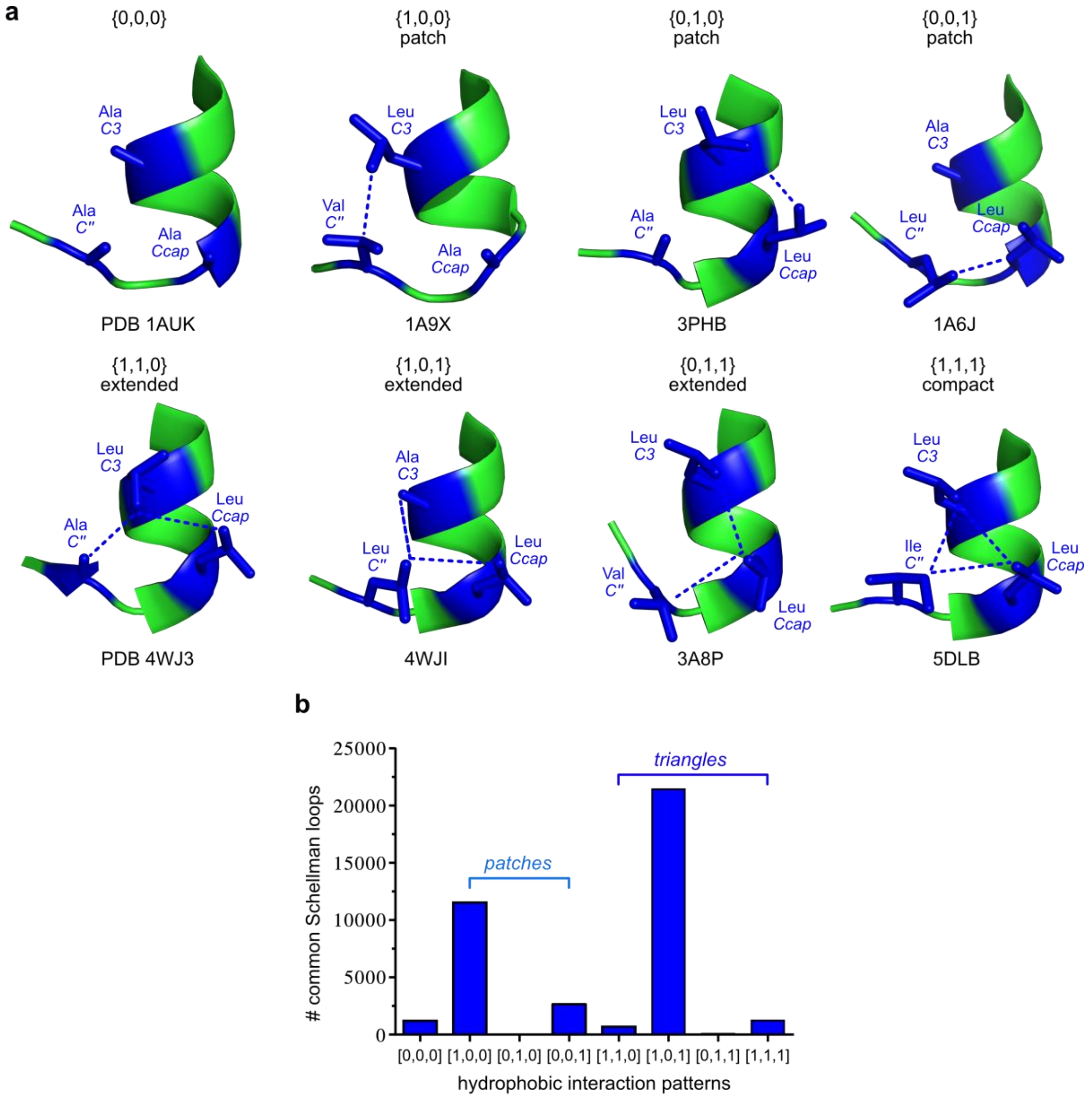

Fig S7 a. illustrative crystal structures of 8 patterns. **b.** Distribution of hydrophobic interaction patterns of α-helical common Schellman loops.

In α-helical common Schellman loops, triangles are more preferred over patches. Extended forms, especially {1,0,1}, are dominant among triangles. This probably indicates the *C3-Ccap* interaction are less likely to happen unless most appropriate residues are placed in *C3* and *Ccap*. Therefore, a detailed study on preference of residue combinations at (*C3*,*Ccap, C''*) in different hydrophobic patterns was made to better understand the inner relationships.

Residues Preference at (*C3*, *Ccap*, *C''*) in Different Hydrophobic Patterns

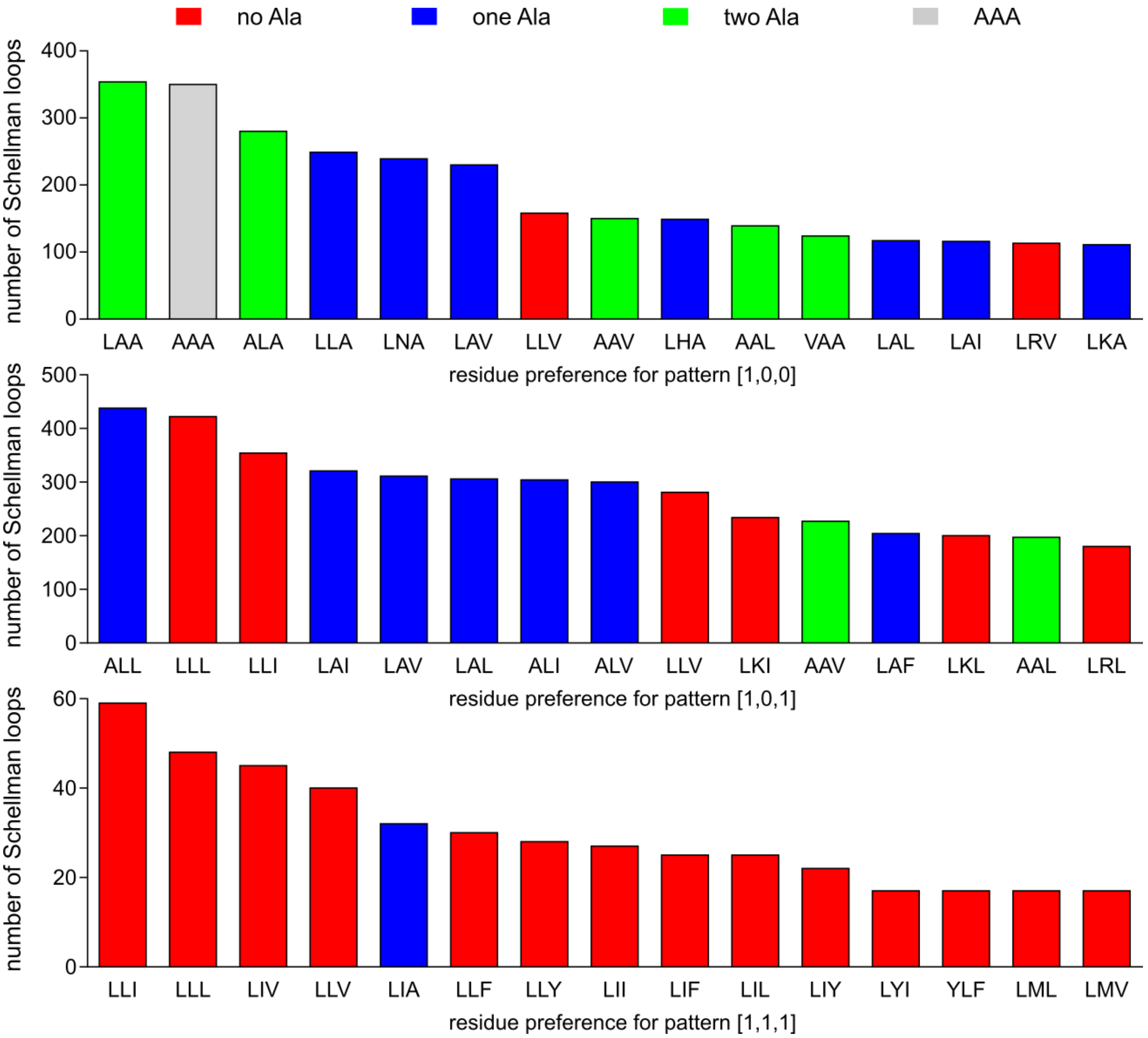

Fig S8 Frequencies of representative patterns: {1,0,0}, {1,0,1} and {1,1,1} for different types of residue trios in helical common Schellman loops.

Residue trios in the sequence are represented as (*C3CcapC''*), such as LLI and LLL, and they are differentiated by colors: a trio with *three* Ala is grey, with *two* Ala is green, with *one* Ala is blue, and *without* Ala is red. Since Ala has shortest sidechains (besides Gly), it is less likely to

participate in the sidechain hydrophobic interactions unless in close distance with another sidechains. As a result, from {1,0,0} to {1,1,1}, there are less Ala in the sequence. Longer aliphatic sidechains from L and I gradually take the leads when more interactions occur.

How many hydrophobic triangles are at surfaces of parent proteins (chains)?

Using 23509 nonredundant hydrophobic triangles found in previous sections, a script was made in house to analyze if they were at the surfaces of the parent chains. Residues at interfaces of multiple chains are still at the surface of the chain they belong to. This is achieved by analyzing the solvent accessible surface area of *C3*, *Ccap* and *C''* that form hydrophobic triangles. If the area of any residue is larger than 5 Å^2, than that residue is regraded as an surface residue. 5 Å^2 is set as the cutoff so that those mostly buried residues with little surface area can still be regarded as buried.

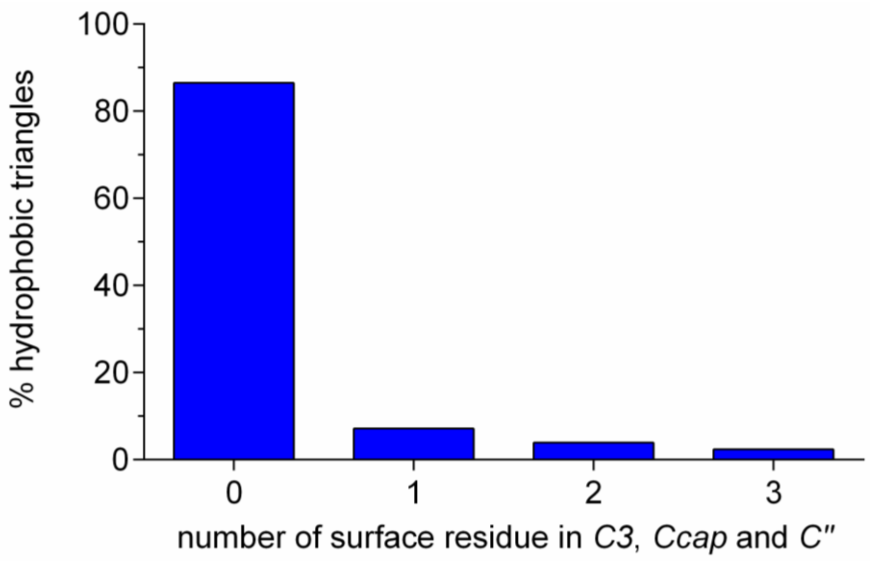

Fig S9 Percent hydrophobic triangles with zero, one, two or three surface residues.

Among 23509 hydrophobic triangles, 87% are completely buried, and only 2% are completely at the surface. This indicates that hydrophobic triangles are preferred to be buried inside proteins. Once buried, they are unlikely to be isolated hydrophobic islands surrounded by hydrophilic oceans: this is not stable. Instead, they are more likely to be a part of hydrophobic network and interact with nearby hydrophobic fragments to stabilize each other.

Search of Hydrophobic Patch and Triangle In *C-cap* Wide Schellman Loops

Wide Schellman Loops at α-helix *C*-termini

Among 2644 wide Schellman loops at α-helical *C*-cap, 1310 which had hydrophobic residues at *C4* and *C''* were used in this study. The calculation used the similar scripts as above. The only difference is *C4* was used instead of *C3* for wide type motifs.

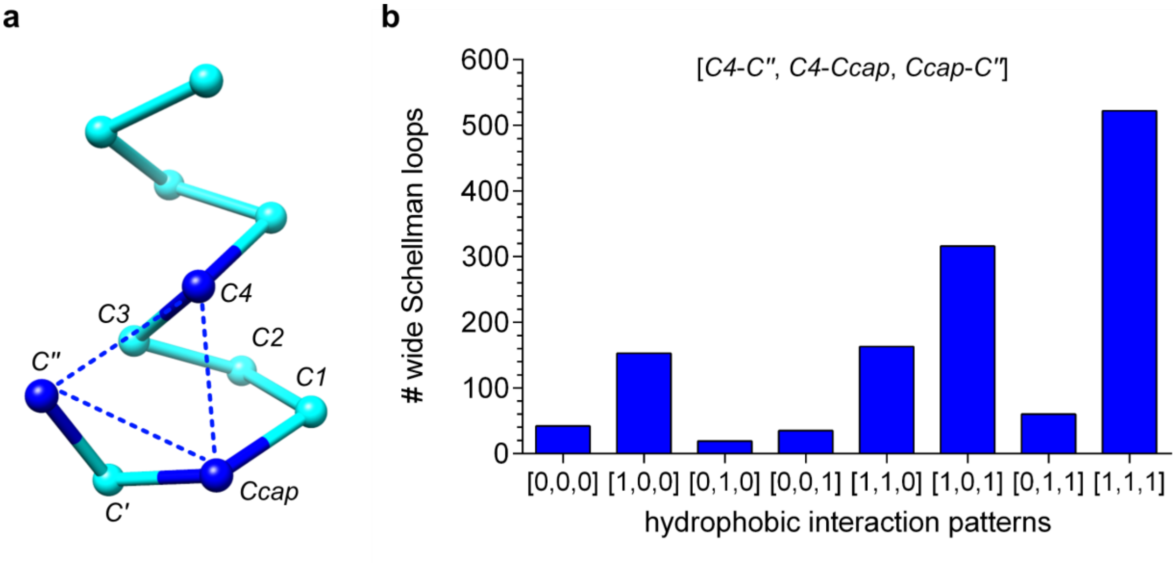

Fig S10 a. interactions between side chains of residues *C4*,*Ccap* and *C''* to form hydrophobic triangles. **b**. hydrophobic interaction patterns of α-helical wide Schellman loops.

Compared with α-helical common Schellman loops, wide Schellman loops are much more inclined to form compact hydrophobic triangles. In common Schellman loops, extended triangles, {1,0,1} are the most abundant, while here compact triangles {1,1,1} takes the lead. It probably indicates the spatial conformation of wide types are more favorable to form hydrophobic triangles than common types.

Wide Schellman Loops at π-helix *C*-termini

Among 1132 wide Schellman loops at π-helical *C*-cap, 582 which had hydrophobic residues at *C4* and *C''* were used in this study. The calculation used the same scripts as above.

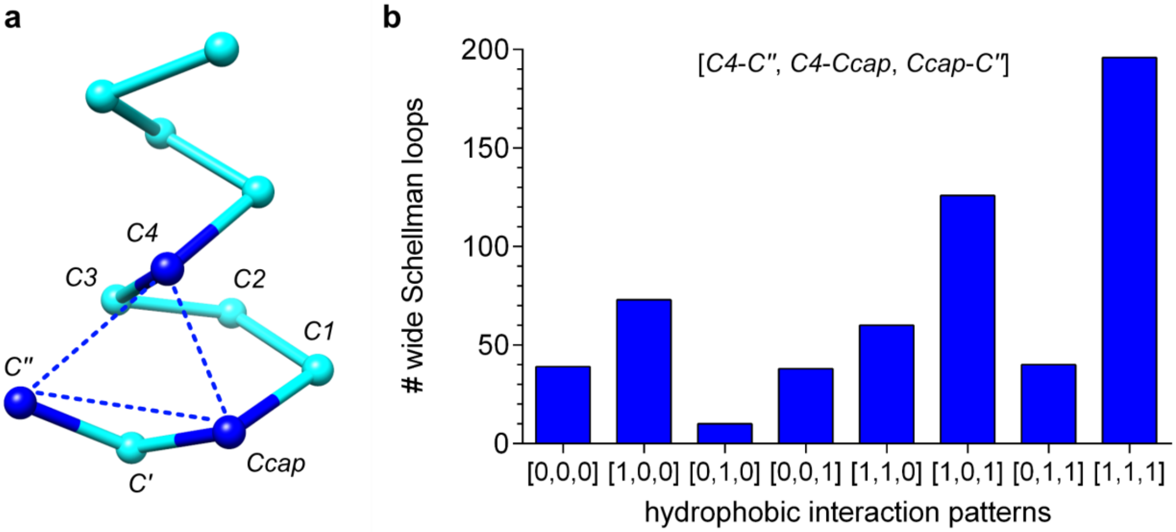

Fig S11 a. interactions between side chains of residues *C4*,*Ccap* and *C''* to form hydrophobic triangles. **b**. hydrophobic interaction patterns of π-helical wide Schellman loops.

Wide Schellman loops at π-helix *C*-termini have similar trends to those at α-helix termini. Here trios with compact triangles {1,1,1} are the most abundant, though slightly less abundant than at α-helix termini, but more so than common Schellman loops. It supports that the spatial conformation of wide types are more inclined to form hydrophobic triangles than common types.

D. Hot Segment Analysis on Interface Schellman Loop Dataset

Hot Interface Schellman Loops

Among all Schellman loops in PDB, there were 104151 at the interface. These 104151 common and wide Schellman loops were the targets in this study. $\frac{\Delta SASA(Schellman\ loop)}{\Delta SASA(chain)}$ and $\frac{\Delta SASA\ (Schellman\ loop)}{number\ of\ residues}$ were calculated for each Schellman loops. If the former is higher than 10% and the latter larger than 50 Å, the Schellman loop is considered as 'hot Schellman loop'.

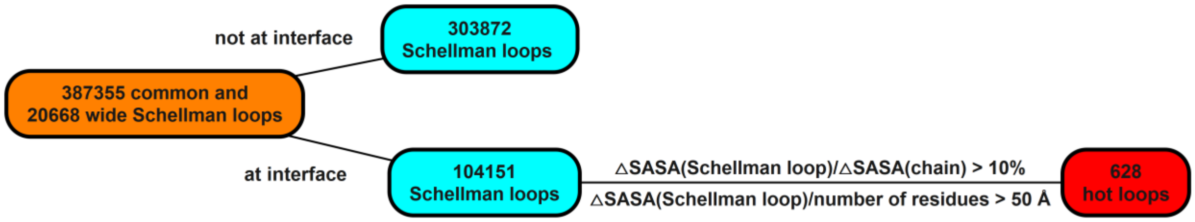

Fig S12 search of hot Schellman loops from all interface Schellman loops.

Among 104151 interface Schellman loops, only 628 (0.6%) are hot loops. Such small ratio indicates that Schellman loops are not likely to provide large interface to bind to another protein receptors, but more likely to be a functional group to end (and propagate) helices at the *C*-termini.

E. Syntheses of Peptides

Linear Peptides

The linear control peptides were synthesized using standard Fmoc peptide synthesis protocols (Scheme S1) on TentaGel S RAM resin (capacity = 0.22 mmol/g) either on a LibertyBlue peptide synthesizer or manually. Couplings were carried out using 5 equivalents (equiv) of Fmoc-protected amino acid, 10 equiv of Oxyma, 10 equiv of DIC. Coupling reactions were allowed to proceed for 2 minutes in DMF at 90 °C, after which Fmoc deprotection was carried out with 20% (vol/vol) piperidine in DMF at 90 °C for 1 minutes. Upon completion of the peptide sequence assembly on resin and deprotection of the final Fmoc group, the *N*-terminal amine was acetylated by treatment with 25% (vol) acetic anhydride and 3.5% (vol) DIPEA in DMF. Cleavage and deprotection from the solid support was carried out using TFA/H2O/TIPS (95/2.5/2.5 vol/vol) for 3 hours at room temperature. The resin was then filtered and washed with excess TFA. The isolated TFA solution was then evaporated, and the peptide was precipitated by adding cold diethyl ether. The peptide suspension in ether was centrifuged to pellet followed by decanting the ether. The pellet was then washed twice with cold diethyl ether. Purification of the resulting peptides was achieved by high performance liquid chromatography (HPLC) on a reversed-phase C8 column to yield peptide with purity >95%. Purity and identity were assessed using ESI-MS and analytical HPLC on a C18 column.

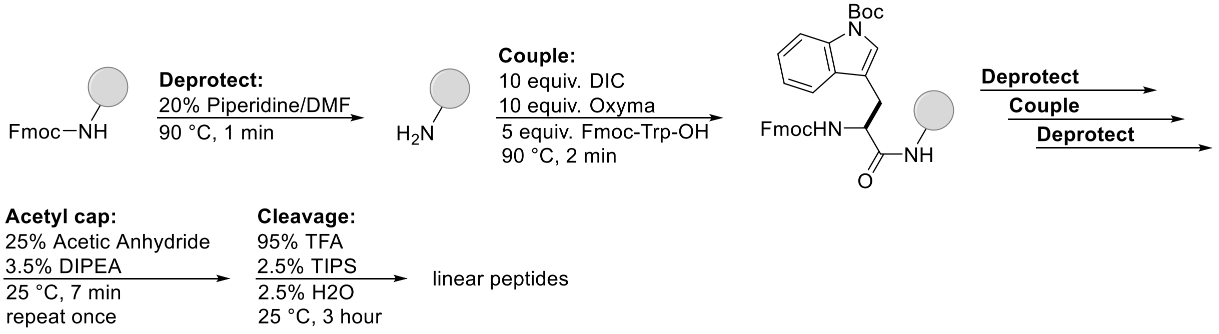

Scheme S1 Synthesis route for linear peptides.

Bicyclic Capped Peptides

The linear peptides were synthesized using standard Fmoc peptide synthesis protocols (Scheme S2) on TentaGel S RAM resin (capacity = 0.22 mmol/g) either on a LibertyBlue peptide synthesizer or manually. The three cystines were placed at *C3*, *Ccap* and *C''* near the *C*-terminus. Couplings were carried out using 5 equivalents (equiv) of Fmoc-protected amino acid, 10 equiv of Oxyma, 10 equiv of DIC. Coupling reactions were allowed to proceed for 2 minutes in DMF at 90 ℃, after which Fmoc deprotection was carried out with 20% (vol/vol) piperidine in DMF at 90 ℃ for 1 minutes. Upon completion of the peptide sequence assembly on resin and deprotection of the final Fmoc group, the N-terminal amine was acetylated by treatment with 25% (vol) acetic anhydride and 3.5% (vol) DIPEA in DMF. Cleavage and deprotection from the solid support was carried out using TFA/H2O/TIPS (95/2.5/2.5 vol/vol) for 3 hours at room temperature. The resin was then filtered and washed with excess TFA. The isolated TFA solution was then evaporated, and the peptide was precipitated by adding cold diethyl ether. The peptide suspension in ether was centrifuged to pellet followed by decanting the ether. The pellet was then washed twice with cold diethyl ether and dissolved in 1:1 mixture of aqueous buffer (20mM NH_4HCO_3) and ACN to make final concentration around 1 mM. 1.5 equiv of TBMB was added and the solution was stirred for 15 min at room temperature. Purification of the resulting peptides was achieved by high performance liquid chromatography (HPLC) on a reversed-phase C8 column to yield peptide with purity >95%. Purity and identity were assessed using ESI-MS and analytical HPLC on a C18 column.

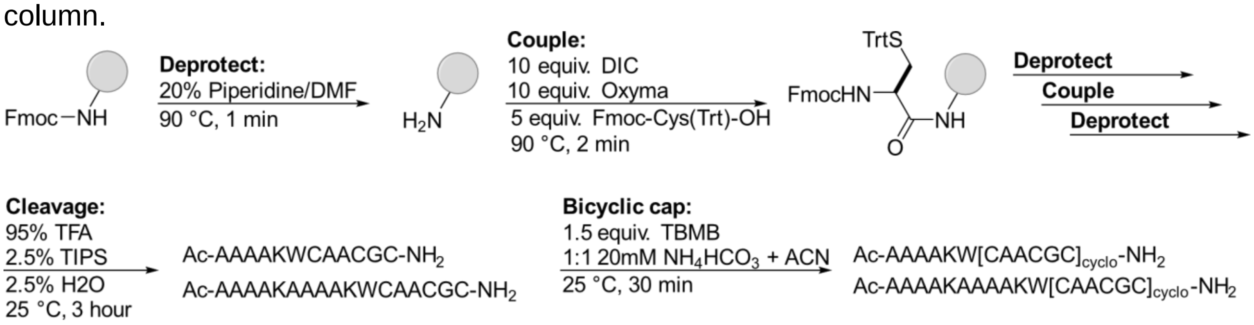

Scheme S2 Synthesis route for bicyclic peptides.

The conversion from linear peptides to bicyclic peptides is both fast (15 min) and clean (only one major peak after cyclization). LCMS curves of the *crude* reactant and product of bicyclo 17-mer is shown in Fig S12. This proved the high accessibility of the new bicyclic caps.

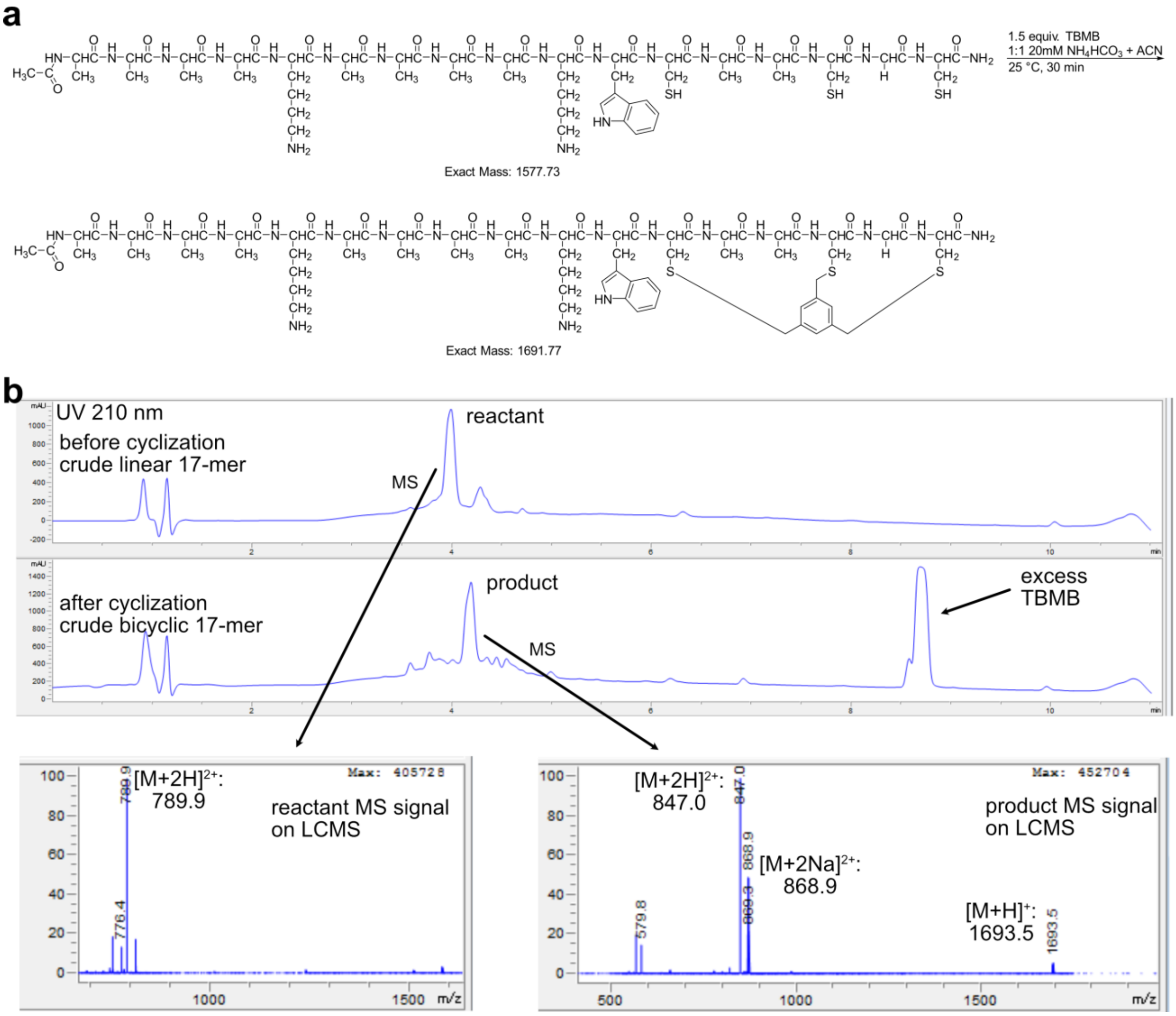

Fig S13 a. linear 17-mer reacted with TBMB to form bicyclo 17-mer. **b**. LCMS traces of *crude* linear 17-mer and bicyclo 17-mer. condition: 70% (0.1% TFA/water)/ 30% (0.1% TFA/acetonitrile) to 5% (0.1% TFA/water)/ 95% (0.1% TFA/acetonitrile) in 9 min.

F. Circular Dichroism (CD) Experiments

Concentrations of the stock solution were determined by 288 nm absorption of Trp and/or Tyr (in LLW 17-mer, two Trps were considered; in LLY 17-mer, Trp and Tyr were considered). Each sample was dissolved in PBS (pH 7.4, 10 mM) or TFE/PBS (1:4) with the final concentration as 10 μM. CD spectra were acquired using circular dichroism spectrometer (Chirascan) equipped with a temperature controller using 1 mm cell at a scan speed of 0.5 nm/sec at indicated temperature. Each sample was scanned three times and the averaged spectrum was smoothed.

Calculation of percent helicity

For each peptide, mean residue ellipticities ([θ]/(c*n), c = concentration of the peptide, n = number of amino acids in the peptide) were calculated and used in graphics. Percent helicity was calculated based on the equation: helicity % = $[\theta]_{222}/[\theta]_{max}$, where $[\theta]_{max} = (-44000 + 250T)(1 - k/n)]$ (n = number of amino acids in the peptide, T = 25.0 ℃). According to Baldwin's work[5,6], k refers to 'the number of non-*H*-bonded peptide carbonyls in a carboxyamidated peptide when it is completely helical, hence for linear peptide, k was 3, and for bicyclic peptides, k was 4 because of the presence of a Schellman loop at the *C*-terminus, as illustrated in Fig S13.

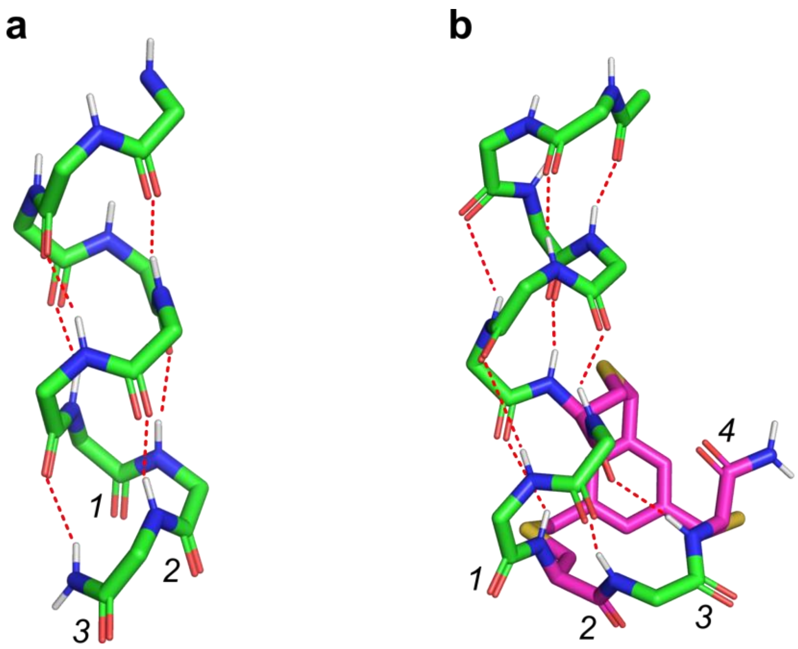

Fig S14 In ideal helical conformations, **a.** linear peptides have 3 non-H-bonded peptide carbonyls, while **b.** bicyclic -capped peptides have 4 (magenta represent three Cys and TMB segment).

CD spectra of synthesized 17-mer linear peptides in PBS buffer

Peptides were synthesized, dissolved in PBS buffer (pH 7.4, 10 mM) and tested at 25 °C. CD curves were shown on the left-hand side and calculated % helicities on the right-hand side.

a **b**

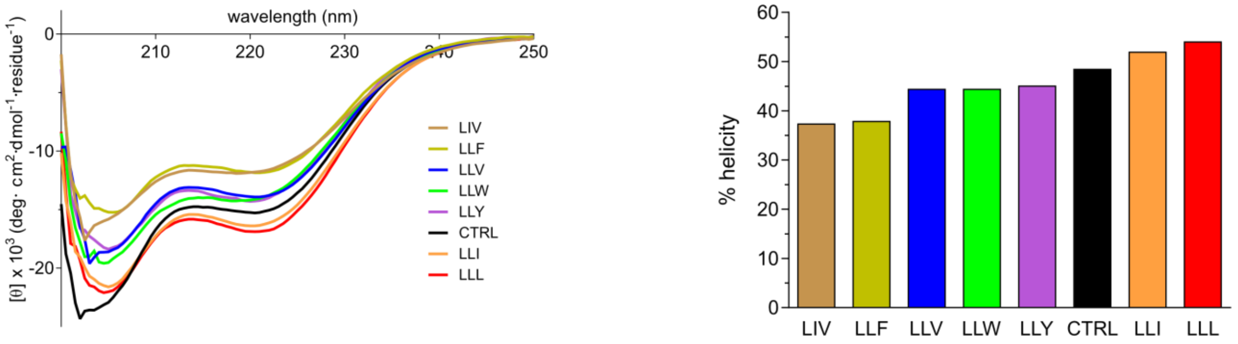

Fig S15 a. processed CD curves of tested linear peptides, including the control and other peptides with different hydrophobic amino acids at *C3*, *Ccap* and *C''*. **b.** corresponding % helicities of linear peptides.

Table S1. Helicities of the synthesized linear 17-mers in PBS buffer.

peptide sequence (label)	solvent	% helicity	$\theta_{222}/\theta_{205}$
Ac-AAAAKAAAAKAAAAKAW-NH$_2$ (CTRL 17-mer/AAKA 17-mer)	PBS	48	0.66
Ac-WAAAKAAAAKA**L**AA**I**G**V**-NH$_2$ (LIV 17-mer)	PBS	37	0.73
Ac-WAAAKAAAAKA**L**AA**L**G**F**-NH$_2$ (LLF 17-mer)	PBS	38	0.77
Ac-WAAAKAAAAKA**L**AA**L**G**V**-NH$_2$ (LLV 17-mer)	PBS	44	0.74
Ac-WAAAKAAAAKA**L**AA**L**G**W**-NH$_2$ (LLW 17-mer)	PBS	44	0.71
Ac-WAAAKAAAAKA**L**AA**L**G**Y**-NH$_2$ (LLY 17-mer)	PBS	45	0.76
Ac-WAAAKAAAAKA**L**AA**L**G**I**-NH$_2$ (LLI 17-mer)	PBS	52	0.75
Ac-WAAAKAAAAKA**L**AA**L**G**L**-NH$_2$ (LLL 17-mer)	PBS	54	0.76

Trp was intentionally moved to the front in those linear peptides with hydrophobic residues to avoid extra interactions among Trp, *C3*, *Ccap* and *C''*. Comparing with the control, helicities of modified linear peptides had some changes, but not significant. All of them had slightly higher $\theta_{222}/\theta_{205}$ and some had slightly higher % helicities. This implies that simply adding hydrophobic residues at *C3*, *Ccap* and *C''* on linear peptides only had limited effect.

CD spectra of synthesized 17-mer bicyclic peptides in PBS buffer

Bicyclic 17-mer was synthesized, dissolved in PBS buffer (pH 7.4, 10 mM) and tested at 25 °C. In order to test if the Gly at *C'* is still crucial for helicity in the bicyclic system, a negative control peptide (bicyclic-G/A 17-mer) was also synthesized and tested. CD curves were shown on the left-hand side and calculated % helicities on the right-hand side.

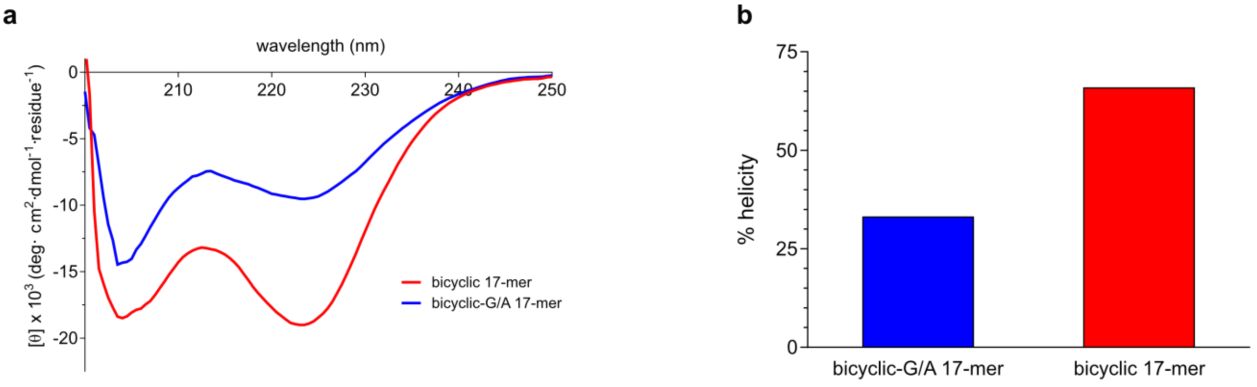

Fig S16 a. processed CD curves of tested bicyclic 17-mers, including the negative control with Ala at *C'*. **b.** corresponding % helicities of bicyclic 17-mers.

Table S2. Helicities of the synthesized linear 17-mers in PBS buffer.

peptide sequence (label)	solvent	% helicity	$\theta_{223}/\theta_{204}$
Ac-AAAAKAAAAKW***C***AA***C***G***C***-NH_2 (bicyclic 17-mer)	PBS	65	1.03
Ac-AAAAKAAAAKW***C***AA***CAC***-NH_2 (bicyclic-G/A 17-mer)	PBS	33	0.66

Comparing to linear peptides, similar trends to the 12-mer series appeared in this 17-mer series: bicyclic 17-mer is more helical than linear ones considering both % helicities and $\theta_{223}/\theta_{204}$ (1.25 ~ 1.75 for ideal canonical α-helix). The huge drop of helicity (32%) from bicyclo 17-mer to bicyclic-G/A 17-mer indicated that Gly at *C'* is crucial and required for maximal function of the synthetic cap.

CD spectra of synthesized 12-mer peptides in 20% TFE/PBS buffer

The four synthesized 12-mers (AAKA 12-mer, [*C3-Ccap*] 12-mer, [*C3-C''*] 12-mer and bicyclo12-mer) were dissolved in 20% TFE/PBS buffer (pH 7.4, 10 mM) and tested at 25 °C. CD curves were shown on the left-hand side and calculated % helicities on the right-hand side.

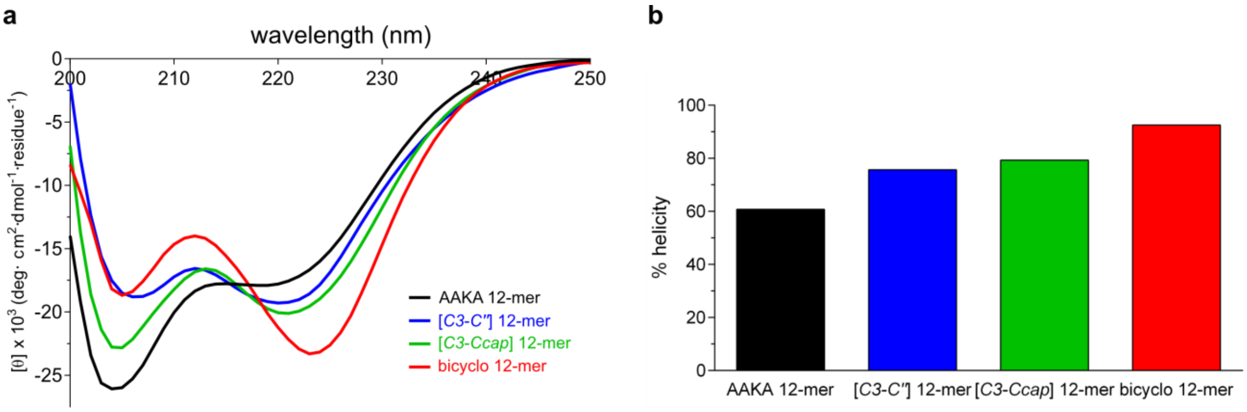

Fig S17 a. processed CD curves of tested 12-mers. **b.** corresponding % helicities of the 12-mers.

Table S3. Helicities of the synthesized 12-mers in 20% TFE/PBS buffer.

peptide sequence (label)	solvent	% helicity	$\theta_{223}/\theta_{206}$
Ac-AAAAKAAAAKAW-NH$_2$ (AAKA 12-mer)	20% TFE/PBS	61	0.66
Ac-AAAAKW***C***AA***C***GA-NH$_2$ ([*C3-Ccap*] 12-mer)	20% TFE/PBS	79	0.86
Ac-AAAAKW***C***AAAG***C***-NH$_2$ ([*C3-C''*] 12-mer)	20% TFE/PBS	76	1.01
Ac-AAAAKW***C***AA***C***G***C***-NH$_2$ (bicyclo 12-mer)	20% TFE/PBS	93	1.23

Helicities of 12-mers were all improved in 20% TFE/PBS, but bicyclo 12-mer is still the most helical one among four peptides. In 20% TFE/PBS, Both % helicities and $\theta_{223}/\theta_{206}$ (1.25 ~ 1.75 for ideal canonical α-helix) indicates that bicyclo 12-mer are close to ideal α-helices.

Variable Temperature CD Experiment

Concentrations of the stock solution were determined by 288 nm absorption of Trp. Each sample was dissolved in PBS buffer (pH 7.4, 10 mM) with the final concentration as 10 μM. CD spectra were acquired using circular dichroism spectrometer (Chirascan) equipped with a temperature controller using 1 mm cell at a scan speed of 2 nm/sec at indicated temperature. Each sample was scanned three times and the averaged spectrum was smoothed. Temperature range (°C) between 5 to 85 with a step of 10 was used in the study. Mean residue ellipticity were calculated and used to make graphics.

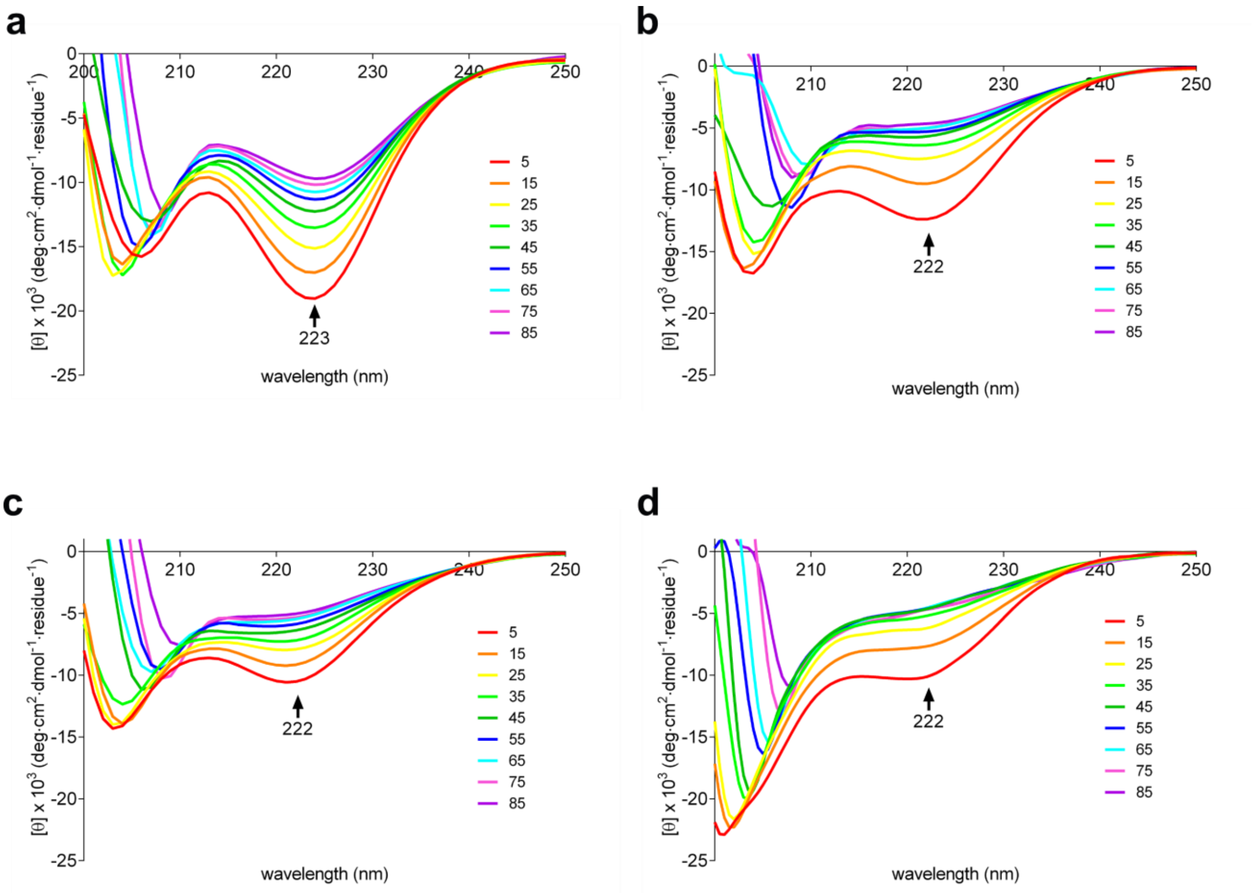

Fig S18 Processed CD curves in temperatures from 5 to 85 °C for **a.** bicyclo 12-mer, **b.** [*C3-Ccap*] 12-mer, **c.** [*C3-C”*] 12-mer and **d.** AAKA 12-mer in PBS buffer.

G. MD on Modeled Bicyclic *C*-capped 12mer

To test if bicyclic skeletons on *C*-termini of peptides can constrain and stabilize conformations of Schellman loops, a model peptide with 12 residues (Ac-AAAAAA*C*AA*C*G*C*, *C* for cyclized Cys) was prepared virtually. In this peptide, 6 Ala composed of the *N*-terminus and the left 6 residues, along with TMB, composed of the bicyclic *C*-cap to mimic the natural Schellman loop. The calculation was made in *Schrödinger* software. The peptide was solvated using *System Builder*, and then put into *Molecular Dynamics* for 1 μs and 4000 frames were kept during the simulation. For each frame, the backbone atoms (*N*, *C*, *O*, *C*α) of the first 6 residues were overlaid with the input atoms, and that RMSD set indicated how the *N*-terminus changed during the simulation period. Similarly, the backbone atoms (*N*, *C*, *O*, *C*α) of the last 6 residues were overlaid with the input atoms to indicate how the bicyclic *C*-cap changed.

H. NMR Experiments

1D and 2D NMR Spectroscopy

Spectra of bicyclo 12-mer (sample prepared in 30 μL D_2O, 120 μL TFE-d_3 and 450 μL H_2O to make final concentration of 2 mM) were recorded on a Bruker AVANCE 500 with a cold probe at 33 °C. Water suppression was achieved by water gate pulse sequence with gradients for 1D ^1H spectrum and by presaturation during relaxation delay for 2D ^1H-^1H spectra. 2D ^1H-^1H TOCSY spectrum was acquired with a mixing time of 80 ms, and ROESY spectrum with a mixing time of 200 ms. The spectra were acquired with 12 ppm spectra width and 2048 × 256 complex points. TOCSY spectrum was processed by TopSpin® to 2048 × 2048 complex points whereas ROESY spectrum to 2048 × 1024 complex points. $^3J_{NH\text{-}H\alpha}$ coupling constants were measured from 1D ^1H spectra and 2D ^1H-^1H TOCSY spectrum.

Bicyclo 12-mer

NMR Spectra

Ac-A1-A2-A3-A4-K-W-[C1-A5-A6-C2-G-C3]$_{cyclo}$

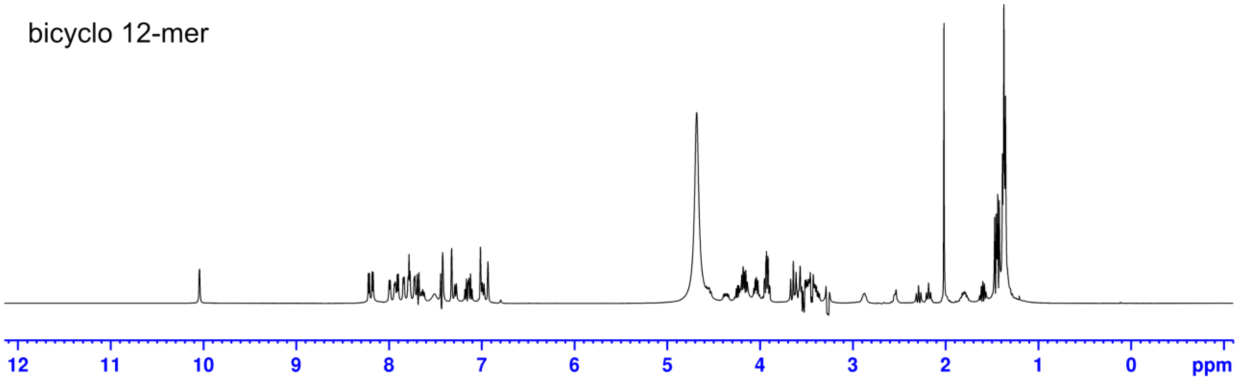

Fig S19 1D H^1 NMR spectrum of bicyclo 12-mer.

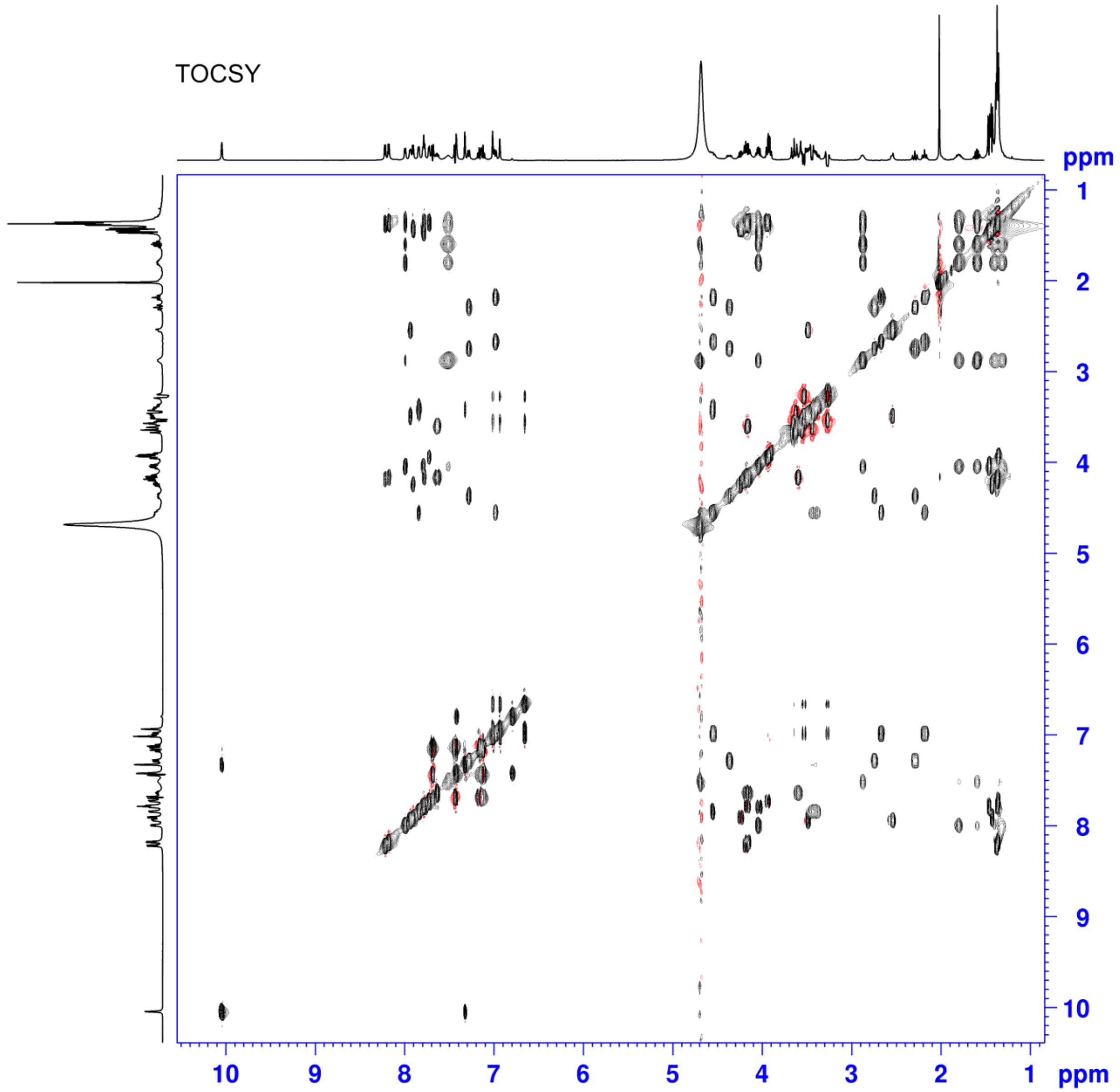

Fig S20 2D H^1-H^1 TOCSY spectrum of bicyclo 12-mer.

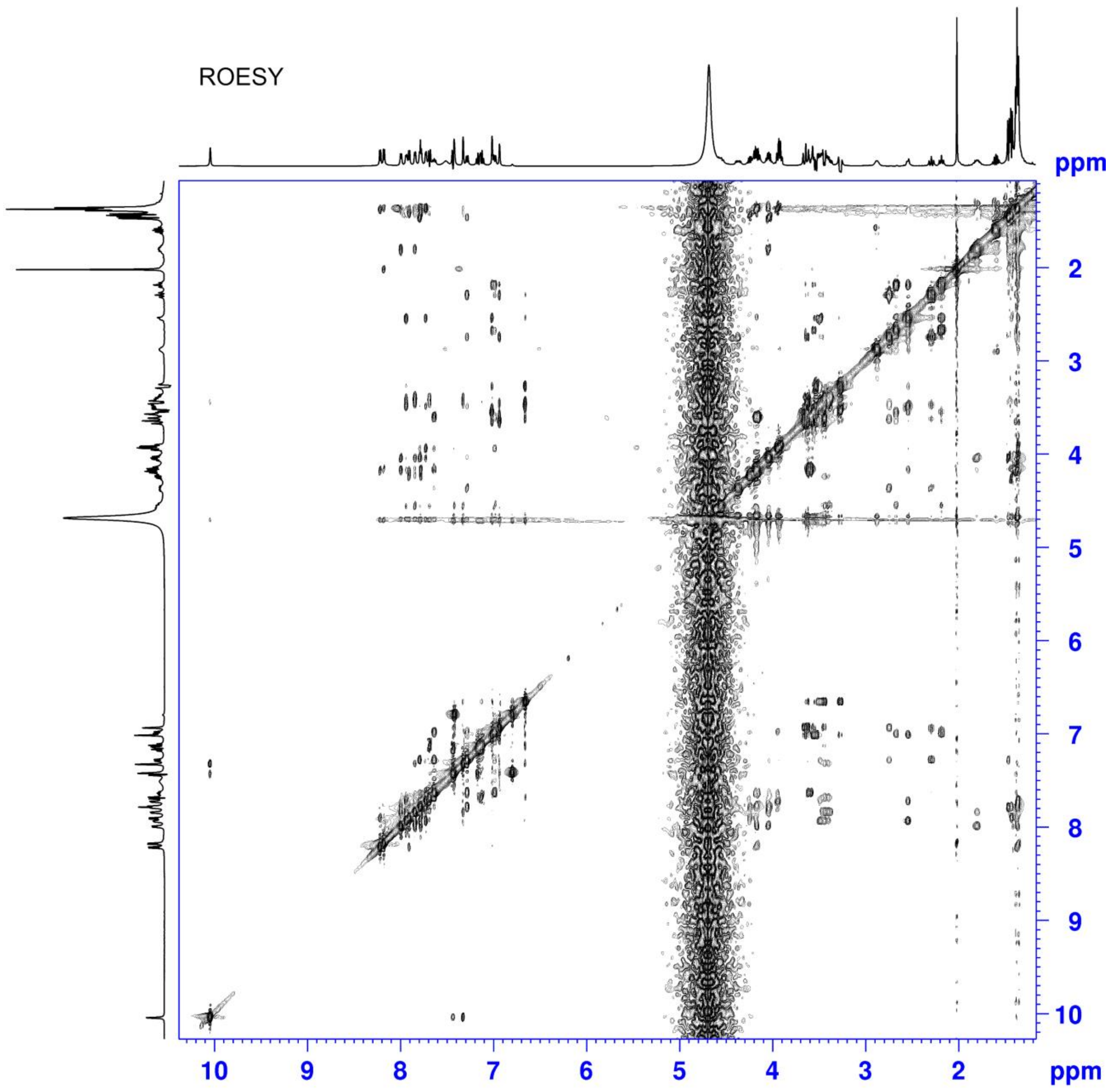

Fig S21 2D ROESY (bottom) spectrum of bicyclo 12-mer

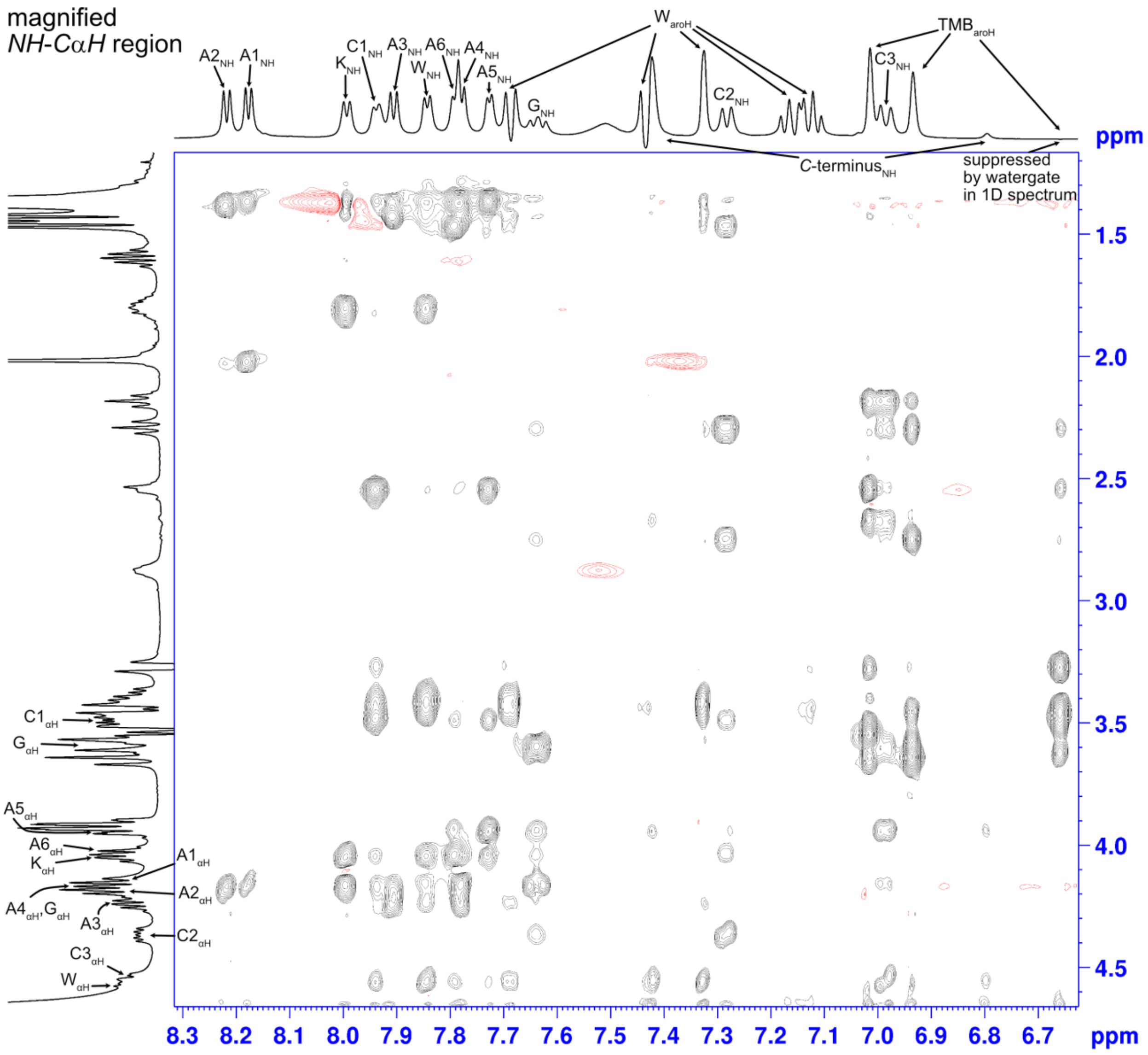

Fig S22 magnified *NH-CαH* fingerprints regions of bicyclo 12-mer from the ROESY spectrum.

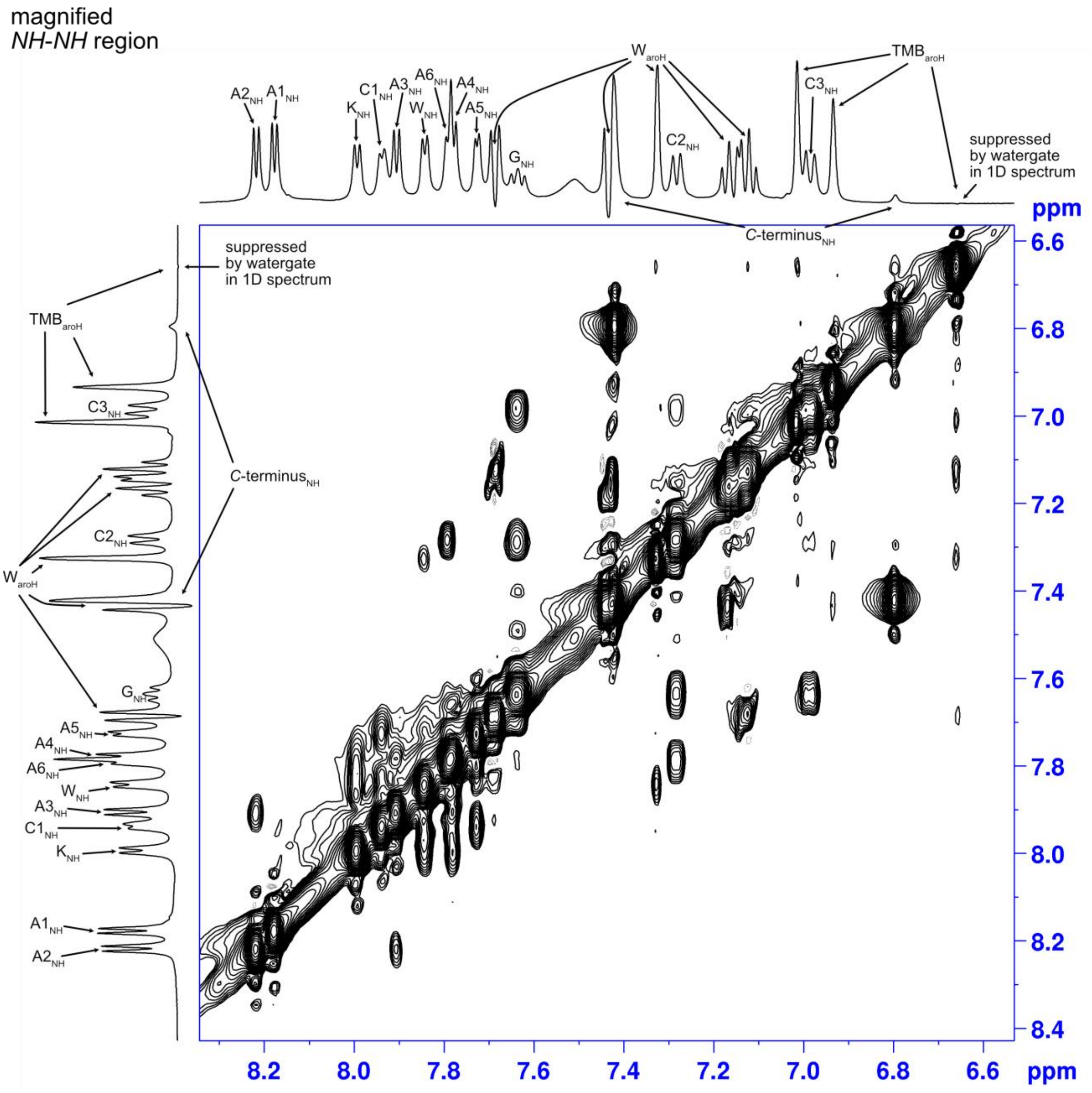

Fig S23 magnified fingerprints regions of LDLL 12-mer from the ROESY spectrum.

Peak Assignment

Ac-A1-A2-A3-A4-K-W-[C1-A5-A6-C2-G-C3]$_{cyclo}$-NH_2

Table S4 Peak assignments for all *H* in bicyclo 12-mer and coupling constants between *NH* and α*H*

Residue	αH	βH	γH	δH	εH	εNH	NH	Aromatic	$^3J_{NH\text{-}\alpha H}$ (Hz)
Ac-	2.02								
A1	4.16	1.36					8.18		5.4
A2	4.19	1.38					8.22		5.7
A3	4.24	1.43					7.91		5.9
A4	4.17	1.36					7.78		5.4
K	4.04	1.80	1.32, 1.40	1.60	2.88	7.51	7.99		5.7
W	4.56	3.40, 3.44					7.84	7.32, 7.43, 7.69, 7.16, 7.13, *N-H*: 10.04	5.2
C1	3.49	2.54					7.93		5.0
A5	3.94	1.36					7.73		4.1
A6	4.03	1.46					7.79		4.9
C2	4.37	2.29, 2.74					7.28		8.2
G	3.60, 4.17						7.64		
C3	4.55	2.18, 2.67					6.98		9.4
-NH_2							6.80, 7.42		
TMB	αH: 3.27, 3.53; 3.44, 3.62; 3.55, 3.66							6.66, 6.94, 7.02	

Distance and Dihedral Constraints

NOEs in the ROESY spectrum were assigned to generate distance restraints as described[7]. $^3J_{NH\text{-}H\alpha}$ coupling constants were measured from 1D ^{1}H spectra and 2D ^{1}H-^{1}H TOCSY spectrum to generate dihedral constraints. A total of 175 distance restraints (summarized in Table S2) and 11 dihedral constraints (in Table S3) were then utilized for structure calculation using Macromodel package in *Schrödinger*. An optimized conformational sampling method, Enhanced MCMM[8], was used to exhaust possible conformations of the input structure and those unmatched conformations were filtered out by the constraints. 71 conformers within 3 kcal/mol (12.6 kJ/mol) to the lowest-energy conformer were then interpreted as the solution structural ensemble.

Table S5 Distance constraints from ROESY spectrum

Atom1	Chemical shift	Atom2	Chemical shift		Upper limit	Lower limit
**Within Residue**						
**A1**						
A1NH	8.1734	A1α	4.1609		3.91	2.61
A1NH	8.1763	Ac-α	2.016		3.47	2.32
A1NH	8.1763	A1β	1.3655		3.57	2.38
**A2**						
A2α	4.1895	A2β	1.3773		3.01	2.00
A2NH	8.2233	Ac-α	2.0219		4.65	3.10
A2NH	8.2233	A2β	1.3773		3.29	2.19
**A3**						
A3α	4.2424	A3β	1.43		3.05	2.03
A3β	1.4319	A3NH	7.8997		3.10	2.06
A3α	4.2424	A3NH	7.8938		3.19	2.13
**A4**						
A4α	4.169	A4NH	7.7825		2.96	1.98
A4NH	7.7686	A4β	1.3538		3.08	2.05
**K**						
Kβ	1.7957	Kα	4.0437		2.90	1.93
KNH	7.9974	Kα	4.0378		3.31	2.20
KNH	7.9945	Kδ	1.6		5.57	3.71
K γ	1.4143	KNH	8.0051		4.00	2.67
KNH	7.9974	Kβ	1.8109		3.00	2.00
**W**						
W-aro-b	7.6864	Wα	4.5535		4.18	2.79
Wβ1	3.3945	W-aro-b	7.677		3.57	2.38
Wβ2	3.4415	W-aro-b	7.6828		3.45	2.30
W-aro-a	7.3256	Wα	4.5418		4.05	2.70
Wβ2	3.4385	W-aro-a	7.3195		3.75	2.50
Wβ1	3.3916	W-aro-a	7.3195		3.67	2.45
Wβ2	3.4385	Wα	4.5476		3.75	2.50
Wβ1	3.4004	Wα	4.5476		3.64	2.43
WNH	7.8448	W-aro-a	7.3254		3.77	2.51
Wβ1	3.3857	WNH	7.8411		3.03	2.02
Wβ2	3.4503	WNH	7.8293		3.08	2.05
WNH	7.8419	Wα	4.5476		3.70	2.47
W-aroNH	10.048	W-aro-e	7.4309		3.67	2.45
W-aroNH	10.048	W-aro-a	7.3195		3.06	2.04
W-aro-d	7.176	W-aro-e	7.4367		2.98	1.99
W-aro-c	7.1261	W-aro-b	7.6887		2.88	1.92
**C1**						
C1α	3.4767	C1β	2.5552		2.76	1.84

C1α	3.4972	C1NH	7.9407		3.05	2.04
**A5**						
A5α	3.9402	A5β	1.3597		2.74	1.83
A5NH	7.7275	A5α	3.9265		3.30	2.20
A5NH	7.7275	A5β	1.3538		2.86	1.91
**A6**						
A6α	4.037	A6β	1.4593		2.74	1.83
A6NH	7.792	A6α	4.0378		3.18	2.12
A6β	1.4671	A6NH	7.7883		2.96	1.97
**C2**						
C2β1	2.3003	C2β2	2.7427		2.38	1.58
C2β2	2.7491	C2α	4.366		3.23	2.16
C2β1	2.2944	C2α	4.366		3.58	2.39
C2NH	7.2816	C2α	4.3601		3.55	2.37
C2NH	7.2845	C2β2	2.7427		3.59	2.40
C2NH	7.2816	C2β1	2.2915		3.09	2.06
**G**						
Gα2	4.1661	Gα1	3.5983		2.20	1.47
GNH	7.6395	GαA2	4.1784		3.39	2.26
GNH	7.6336	Gα1	3.5983		2.92	1.95
**C3**						
C3β2	2.6641	C3β1	2.186		2.35	1.57
C3β2	2.6729	C3α	4.5476		3.58	2.39
C3β1	2.1888	C3α	4.5535		3.86	2.58
C3NH	6.9853	C3α	4.5359		3.99	2.66
C3NH	6.9824	C3β2	2.6724		3.79	2.52
C3NH	6.9853	C3β1	2.1801		3.13	2.09
**C-terminus amide**						
C-N1	6.8034	C-N2	7.4191		2.02	1.35
**Cross residues**						
**N(i)-N(i+1)**						
A2NH	8.2174	A3NH	7.9055		3.37	2.24
A3NH	7.9064	A4NH	7.7707		2.96	1.97
A4NH	7.7803	KNH	8.011		3.11	2.07
KNH	8.0033	WNH	7.8528		2.92	1.95
C1NH	7.9416	WNH	7.8293		2.84	1.89
A5NH	7.7304	C1NH	7.9407		3.16	2.11
A5NH	7.7304	A6NH	7.8		2.80	1.87
A6NH	7.795	C2NH	7.2785		3.13	2.09
C2NH	7.2845	GNH	7.6418		3.01	2.01
GNH	7.6365	C3NH	6.9913		2.96	1.97
C3NH	6.9824	C-N2	7.4074		4.13	2.75
**N(i)-N(i+2)**						
A4NH	7.7803	A2NH	8.222		5.17	3.45

GNH	7.6395	A6NH	7.7883		4.35	2.90
C2NH	7.2845	C3NH	6.9738		3.57	2.38
**α(i)-N(i+1)**						
A2α	4.1778	A3NH	7.8938		3.46	2.30
A3α	4.2424	A4NH	7.7883		3.53	2.35
WNH	7.8419	Kα	4.0378		3.53	2.35
C1NH	7.9387	Wα	4.5476		4.15	2.77
A5NH	7.7304	C1α	3.4869		3.95	2.63
A6NH	7.7979	A5α	3.9265		4.27	2.85
C2NH	7.2845	A6α	4.0378		4.16	2.78
GNH	7.6395	C2α	4.366		4.14	2.76
C3NH	6.9882	Gα2	4.1609		4.39	2.93
C3NH	6.9794	Gα1	3.5983		3.58	2.39
C-N2	7.4165	C3α	4.5301		3.86	2.58
C-N1	6.8005	C3α	4.5476		4.39	2.93
**α(i)-N(i+2)**						
WNH	7.8448	A4α	4.155		4.22	2.81
C1NH	7.9416	Kα	4.0378		4.29	2.86
A5NH	7.7275	Wα	4.5535		4.75	3.17
A6NH	7.7891	C1α	3.4811		4.58	3.05
C2NH	7.2816	A5α	3.9265		5.25	3.50
GNH	7.6365	A6α	4.0378		4.29	2.86
**α(i)-N(i+3)**						
WNH	7.8448	A3α	4.2488		3.94	2.63
C1NH	7.9416	A4α	4.1667		4.08	2.72
A5NH	7.7304	Kα	4.0495		3.70	2.47
A6NH	7.792	Wα	4.5594		4.51	3.01
C2NH	7.2845	C1α	3.4811		4.09	2.73
GNH	7.6365	A5α	3.9265		3.93	2.62
**α(i)-N(i+4)**						
C2NH	7.2845	Wα	4.5711		5.80	3.87
C3NH	6.9853	A5α	3.9323		3.69	2.46
**α(i)-N(i+5)**						
C-N2	7.4195	A5α	3.944		4.61	3.07
C-N1	6.7975	A5α	3.9323		4.71	3.14
**β(i)-N(i+1)**						
A2β	1.3733	A3NH	7.8997		3.23	2.15
A3β	1.4261	A4NH	7.7825		3.38	2.25
A4β	1.3645	KNH	8.0051		3.25	2.17
WNH	7.8478	Kβ	1.7992		3.36	2.24
Wβ1	3.3887	C1NH	7.9407		3.60	2.40
Wβ2	3.4385	C1NH	7.9465		3.40	2.27
A5NH	7.7246	C1β	2.5434		3.37	2.24
A6NH	7.7979	A5β	1.3538		3.10	2.07
C2NH	7.2845	A6β	1.471		3.60	2.40

GNH	7.6424	C2β2	2.7427		4.42	2.95
GNH	7.6365	C2β1	2.3032		4.35	2.90
C-N2	7.4224	C3β2	2.6782		4.56	3.04
C-N1	6.7975	C3β2	2.6724		5.06	3.37
**Other β-N**						
C2NH	7.2816	C1β	2.5317		5.61	3.74
C3NH	6.9824	C1β	2.5259		4.88	3.25
WNH	7.839	C1β	2.5493		5.17	3.45
C3NH	6.9853	C2β2	2.7661		4.86	3.24
C3NH	6.9853	C2β1	2.2973		4.02	2.68
C2NH	7.2816	C3β1	2.1918		6.21	4.14
**α(i)-β(i+3)**						
Kβ	1.8074	A2α	4.1784		3.86	2.58
Wβ2	3.4503	A3α	4.237		3.57	2.38
Wβ1	3.3945	A3α	4.237		3.99	2.66
C1β	2.5409	A4α	4.1726		3.63	2.42
Kα	4.0458	A5β	1.3421		3.19	2.13
C1α	3.4943	C2β2	2.7368		4.06	2.71
C1α	3.4943	C2β1	2.2856		4.00	2.67
**β-β**						
C3β2	2.6787	C3β2	2.6787		2.6787	C3β2
C1β	2.5379	C1β	2.5379		2.5379	C1β
**W-related**						
WNH	7.8448	Kγ	1.4007		3.34	2.23
W-aro-b	7.6894	A3α	4.237		4.55	3.03
**TMB-related**						
TMB-α1-2	3.5353	TMB-α1-1	3.276		2.31	1.54
C1β	2.5438	TMB-α1-1	3.2701		4.02	2.68
TMB-aro3	7.0146	TMB-α1-1	3.2643		3.91	2.61
TMB-α1-1	3.2596	TMB-aro1	6.6514		2.91	1.94
W-aro-c	7.1261	TMB-α1-1	3.2525		4.38	2.92
TMB-α1-1	3.2684	W-aro-b	7.6946		3.97	2.65
TMB-α1-1	3.2713	C1NH	7.9407		4.45	2.96
TMB-α1-2	3.5383	C1β	2.5376		3.61	2.41
TMB-α1-2	3.5031	TMB-aro3	7.0206		2.78	1.85
TMB-α1-2	3.5383	TMB-aro1	6.6632		3.66	2.44
TMB-α2-1	3.4473	TMB-α2-2	3.6276		2.14	1.43
TMB-α2-1	3.4444	C2β2	2.7368		4.26	2.84
TMB-α2-1	3.4444	C2β1	2.2856		4.19	2.79
TMB-aro2	6.9354	TMB-α2-1	3.4459		3.64	2.43
TMB-α2-1	3.4385	TMB-aro1	6.669		2.88	1.92
W-aro-c	7.129	TMB-α2-1	3.4401		4.02	2.68
W-aro-d	7.1672	TMB-α2-1	3.4518		5.10	3.40
TMB-α2-1	3.4473	W-aro-e	7.4309		4.54	3.02
TMB-α2-1	3.4503	W-aroNH	10.0445		4.28	2.85

TMB-α2-2	3.6322	C2β2	2.7427		3.33	2.22
TMB-α2-2	3.6322	C2β1	2.2915		4.05	2.70
TMB-α2-2	3.6175	TMB-aro2	6.921		2.99	1.99
TMB-α2-2	3.6292	TMB-aro1	6.6573		3.70	2.47
C2NH	7.2845	TMB-α2-2	3.6159		5.36	3.57
C3β2	2.6758	TMB-α3-1	3.5573		3.10	2.07
TMB-α3-1	3.5559	C3β1	2.1801		3.94	2.63
TMB-α3-1	3.5676	TMB-aro3	7.0206		2.74	1.83
TMB-α3-1	3.5559	TMB-aro2	6.921		3.62	2.41
C3β2	2.6729	TMB-α3-2	3.6569		3.90	2.60
TMB-α3-2	3.6498	C3β1	2.1801		4.47	2.98
TMB-α3-2	3.6586	TMB-aro3	7.0324		3.43	2.29
TMB-α3-2	3.6644	TMB-aro2	6.921		3.04	2.03
TMB-aro1	6.6597	C2β2	2.7486		5.53	3.69
C1α	3.4913	TMB-aro1	6.6632		3.21	2.14
C1β	2.5321	TMB-aro1	6.6514		4.29	2.86
C2β1	2.2915	TMB-aro1	6.6632		4.59	3.06
W-aro-d	7.1672	TMB-aro1	6.6573		5.08	3.39
W-aro-c	7.1261	TMB-aro1	6.6456		3.92	2.61
TMB-aro1	6.6567	W-aro-b	7.6887		4.16	2.78
W-aro-a	7.3285	TMB-aro1	6.669		4.52	3.01
TMB-aro1	6.6626	C1NH	7.9348		4.80	3.20
TMB-aro2	6.9354	C2β2	2.7544		3.23	2.15
TMB-aro2	6.9383	C2β1	2.2915		3.42	2.28
TMB-aro2	6.9383	C3β1	2.1684		4.38	2.92
TMB-aro3	7.0176	C3β2	2.6782		3.55	2.36
TMB-aro3	7.0176	C1β	2.5317		3.26	2.18
TMB-aro3	7.0234	C3β1	2.1801		3.57	2.38

Table S6 Coupling constants and corresponding Φ calculated from equation[9] $\{J(\theta) = 6.98 * (\cos(\theta))^2 – (1.38*\cos(\theta)) + 1.72$, where $\theta = |\Phi-60|\}$.

**Residue**	$^3J_{NH-\alpha H}$ **(Hz)**	**Φ (°)**
A1	5.4	-69 ± 30
A2	5.7	-71 ± 30
A3	5.9	-73 ± 30
A4	5.45	-70 ± 30
K	5.7	-71 ± 30
W	5.2	-68 ± 30
C1	5.0	-66 ± 30
A5	4.05	-59 ± 30
A6	4.95	-66 ± 30
C2	8.2	-90 ± 30
G	\	\
C3	9.4	-103 ± 30

NOE Summary

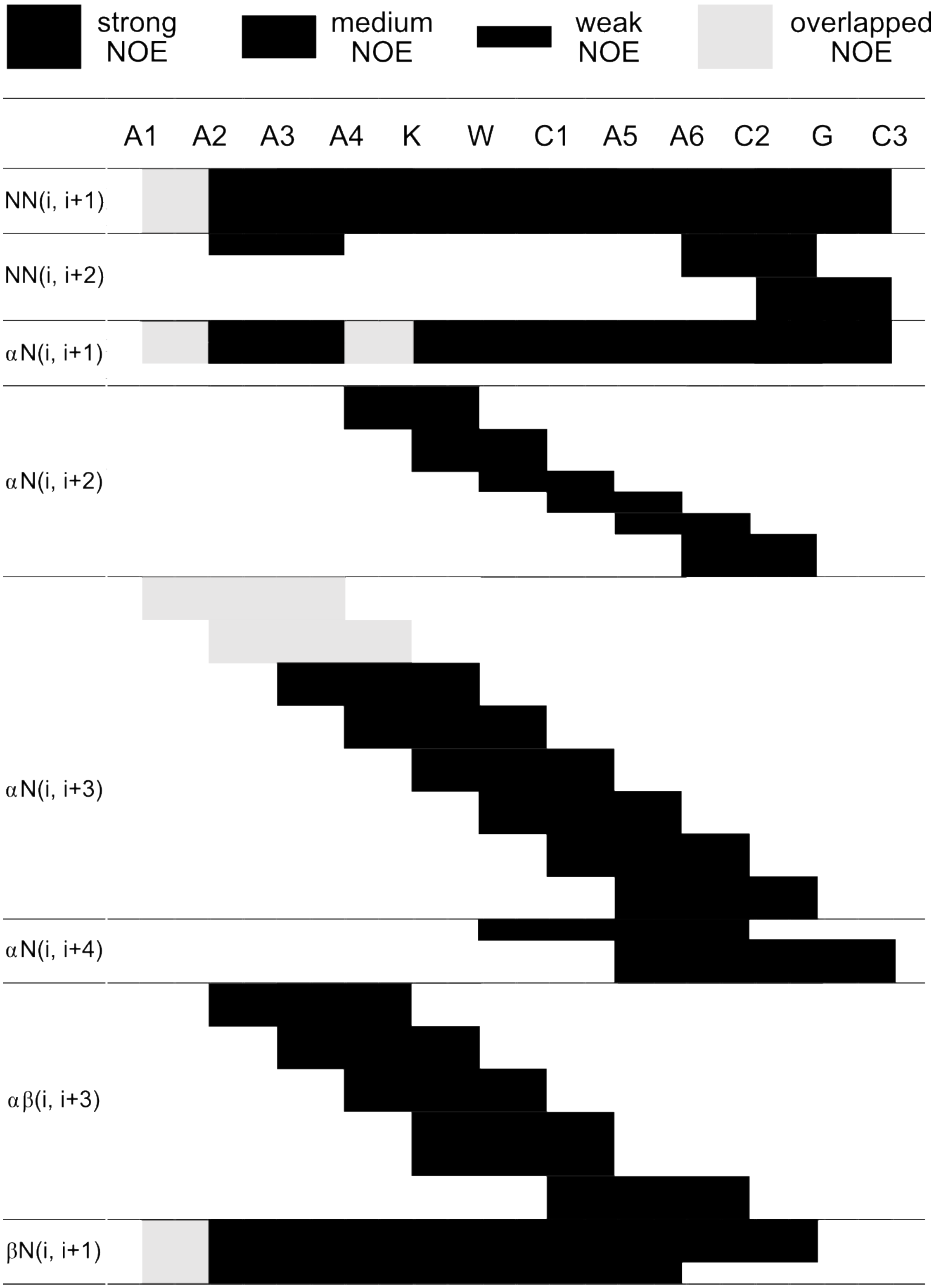

Fig S24 Characteristic cross-residue NOE in bicyclo 12-mer.

Ramachandran Plot of (θ, ψ) Angles

Ac-A1-A2-A3-A4-K-W-[C1-A5-A6-C2-G-C3]$_{cyclo}$-NH_2

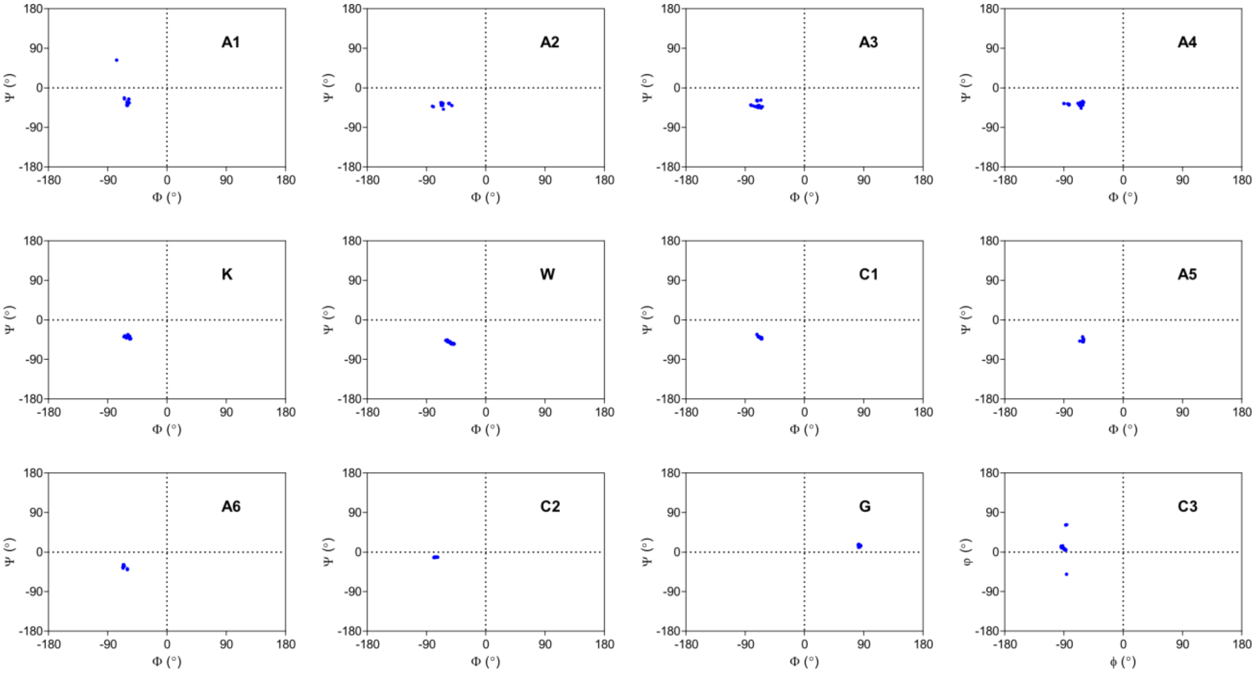

Fig S25 Dihedral angles (Φ,ψ) of residues in low energy conformers of bicyclo 12-mer.

The Impacts of Shielding Effect from TMB to Nearby *H*s

Five *H*s were found to have ‘abnormally’ smaller chemical shifts than their colleagues. Two of them are α*H*s: α*H* of C1 (3.49 ppm) and A5 (3.94 ppm); their chemical shifts are smaller than the other α*H*s. The other three are amide *NH*: *NH* of C2 (7.24 ppm), G (7.64 ppm) and C3 (6.98 ppm); their chemical shifts are the smallest among all amide *NH*s. It can be explained using the conformation derived from NMR constraints. These five *H*s were all in the shielding region of the TMB ring, and the closer to it, the more impact the *H* takes. As a result, three closer *H*s (α*H* of C1, *NH* of C2 and C3) have stronger shielding effect, hence have more decrease in chemical shifts; while α*H* of A5 and *NH* of G are more distant from the benzene ring, hence are less impacted and have smaller decrease in chemical shifts. Fig S24 below describes this observation.

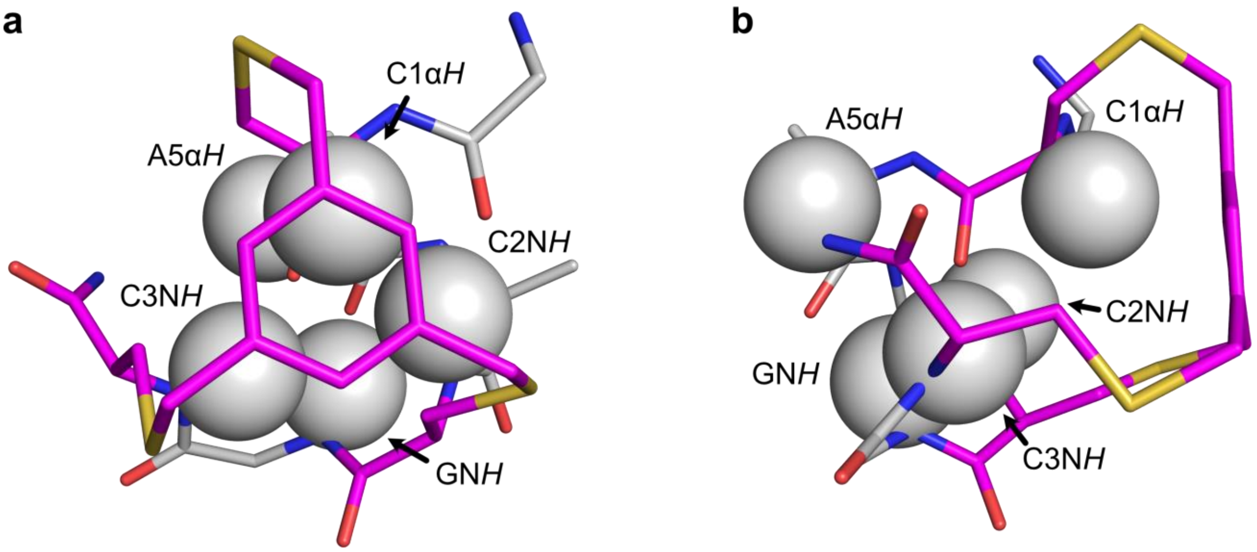

Fig S26 a. Top view of benzene ring and the 5 affected *H*s illustrate they are partially overlapped. **b.** Side view of benzene ring and the 5 affected *H*s showed three are closer to benzene ring than the other two.

Solution Structural Ensembles

Three clusters varied by the *N*-terminus directions.

Cluster 1 (dorminant, 56/71): *N*-terminus stays in helical conformation.
Cluster 2 (minor, 13/71): Ac- works as a cap, forms *O-N H*-bond with A3, similar to a β-turn.
Cluster 3 (minor, 2/71): Ac- forms *O-N H*-bond with A2, similar to an α-turn.

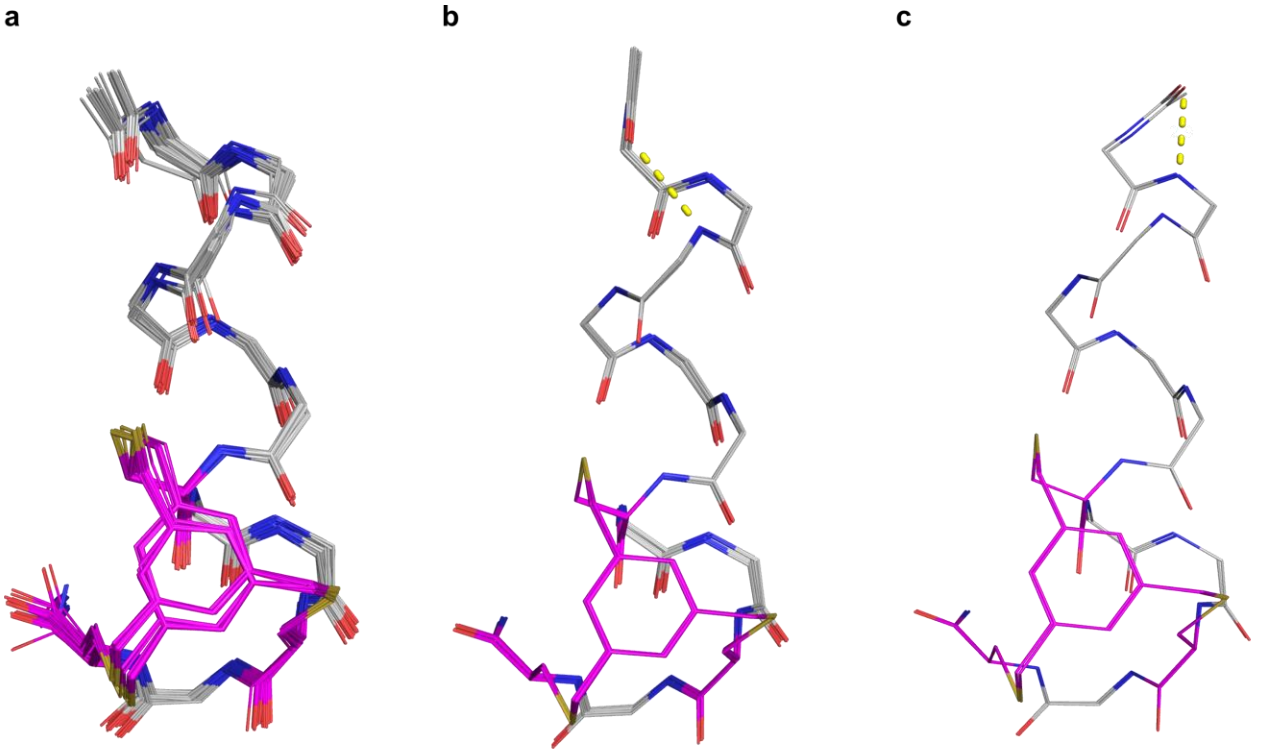

Fig S27 Cluster 1 (left), 2 (middle) and 3 (right) are different from *N*-terminus directions.

Amide H-D Exchange Study

Lyophilized samples of peptide bicyclo 12-mer from the above experiments were dissolved in 600 µL of a D2O/ TFE-d_3 mixture (4:1) to initialize the H-D exchange. The pH of the solution was confirmed. Spectra were recorded on a preshimmed Bruker AVANCE 500 MHz spectrometer. The recorded temperature was 33 °C both inside and outside the probe. The intensity changes for each amide proton were determined by monitoring either the NH peaks on 1D spectra or the cross-peaks between NH and RH on 2D TOCSY spectra when overlapping was severe. The peak intensity data was fit into one phase exponential equation to get the exchange rate constants using GraphPad Prism 6.0 program.

Bicyclo 12-mer H-D Exchange Study

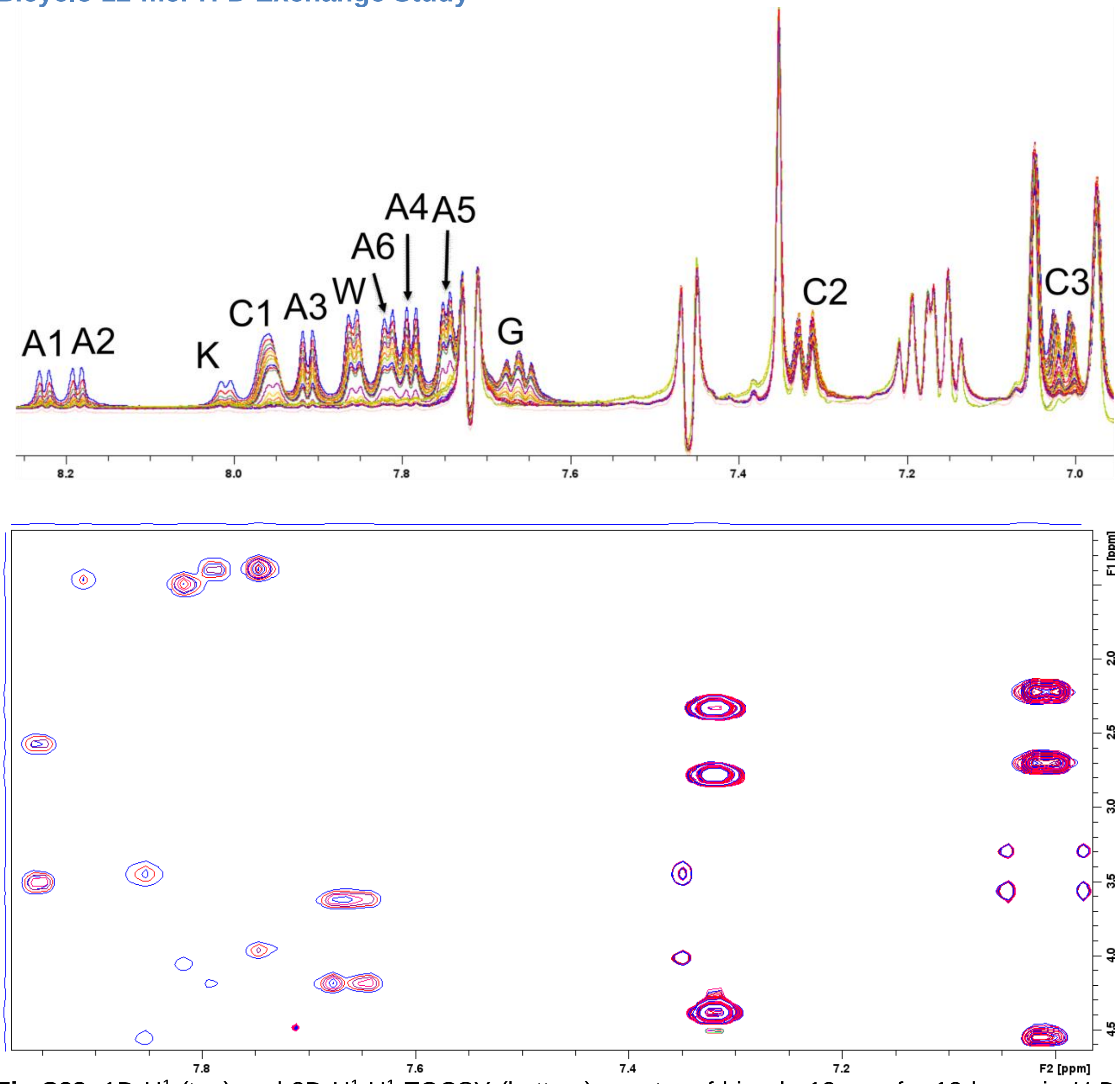

Fig S28 1D H^1 (top) and 2D H^1-H^1 TOCSY (bottom) spectra of bicyclo 12-mer for 12 hours in *H-D* exchange experiments.

Fitting Curves And Summary

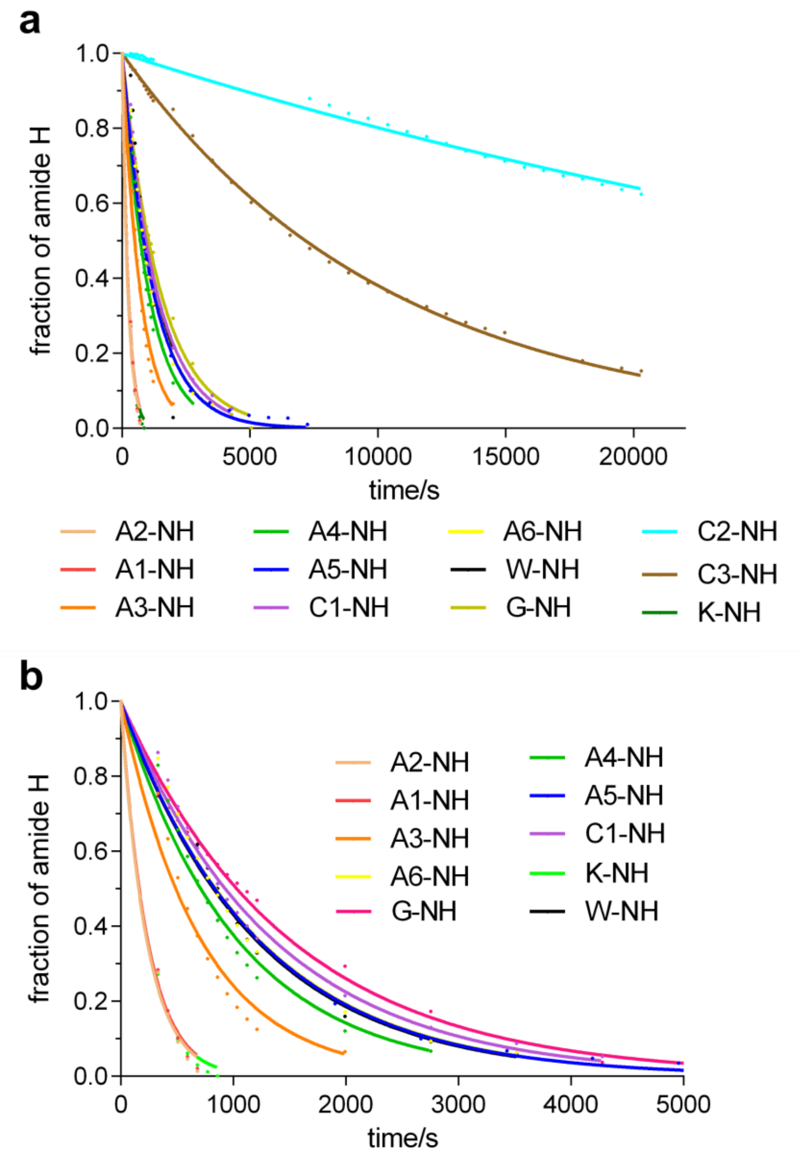

Fig S29 **a.** *H-D* exchange plot for all backbone amide protons in bicyclo 12-mer. **b.** *H-D* exchange plot for backbone amide protons without two extremely stable *H*s from C2 and C3.

Table S7 Summary of *H-D* exchange data[9] for bicyclo 12-mer.

	C9	***C8***	***C7***	***C6***	***C5***	***C4***	***C3***	***C2***	***C1***	***Ccap***	***C'***	***C''***
Bicyclo 12-mer	A1	A2	A3	A4	K	W	C1	A5	A6	C2	G	C3
H/D rate constant × 10^{-4} (s^{-1})	45.54	43.86	14.19	9.76	43.63	8.41	7.47	8.29	8.22	0.22	6.72	0.96
protection factor (log k_{ch}/k_{ex})	1.17	1.35	1.84	2.01	1.31	1.78	2.63	2.63	2.08	4.27	2.69	2.00
stabilization, -ΔG (kcal/mol)	1.59	1.86	2.56	2.79	1.80	2.47	3.67	3.66	2.90	5.96	3.75	2.79

I. Characterization of Purified Peptides

Purity of peptides were measured in analytical HPLC runs using a Zorbax SB-C18 column (Agilent) with a 20 minute gradient between {5% solvent A (99.9% water, 0.1% TFA), 95% solvent B (99.9% acetonitrile, 0.1% TFA)}, and {95% solvent A, 5% solvent B}. Absorption traces at 210 nm were presented along with the retention time for different peptides. Expected masses of peptides were calculated from ChemDraw, and observed masses are $[M+H]^+$ or $[M+2H]^{2+}$ peaks from ESI-MS spectra. Overall yield of each peptide was calculated via a comparison of the amount of substance between the final product (obtained via UV-determined concentration) and resin quantity (0.05 mmol).

AAKA 17-mer $C_{70}H_{116}N_{22}O_{18}$ retention time: 8.215 min calculated $[M+H]^+$: 1553.8916
Observed $[M+H]^+$: 1553.8871 Observed $[M+2H]^{2+}$: 777.4466 overall yield: 35%

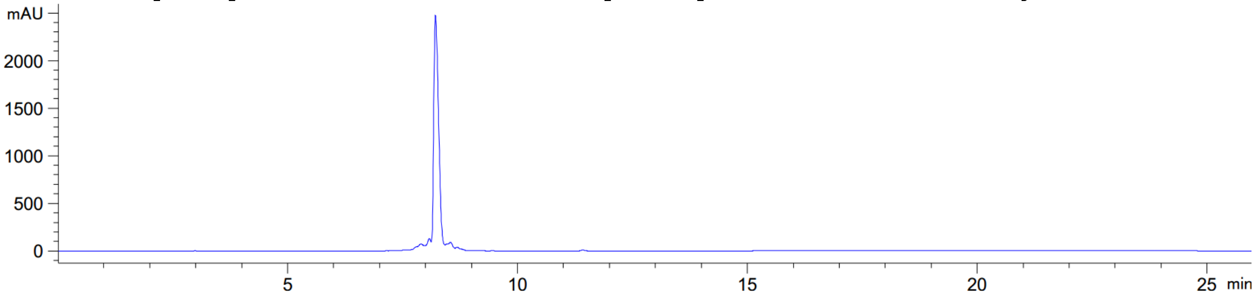

LIV 17-mer $C_{74}H_{123}N_{21}O_{18}$ retention time: 12.841 min calculated $[M+H]^+$: 1594.9433
Observed $[M+H]^+$: 1594.9408 Observed $[M+2H]^{2+}$: 797.9736 overall yield: 30%

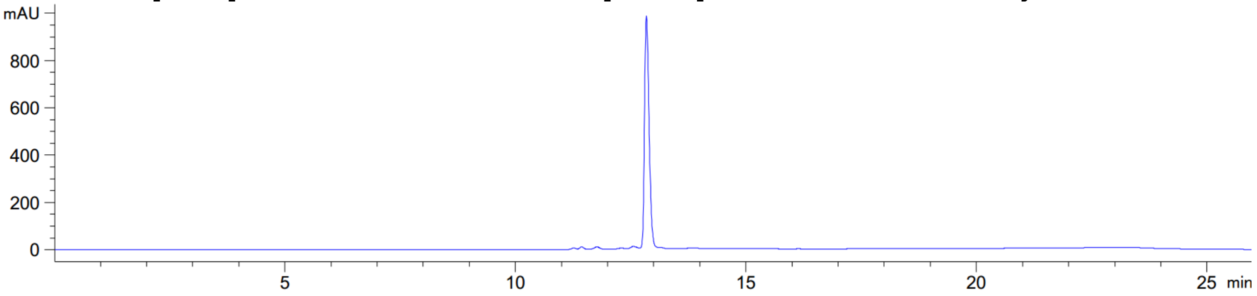

LLF 17-mer $C_{78}H_{123}N_{21}O_{18}$ retention time: 13.760 min calculated $[M+H]^+$: 1642.9433
Observed $[M+H]^+$: 1642.9404 Observed $[M+2H]^{2+}$: 821.9731 overall yield: 39%

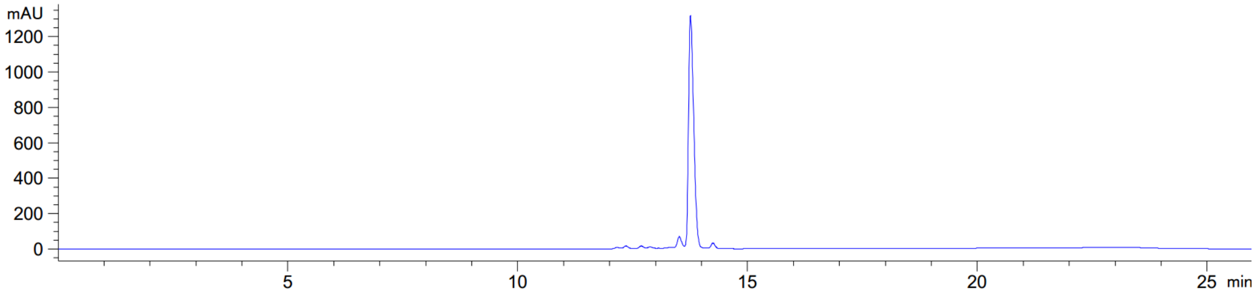

LLV 17-mer $C_{74}H_{123}N_{21}O_{18}$ retention time: 12.995 min calculated $[M+H]^{+}$: 1594.9433
Observed $[M+H]^{+}$: 1594.9405 Observed $[M+2H]^{2+}$: 797.9736 overall yield: 42%

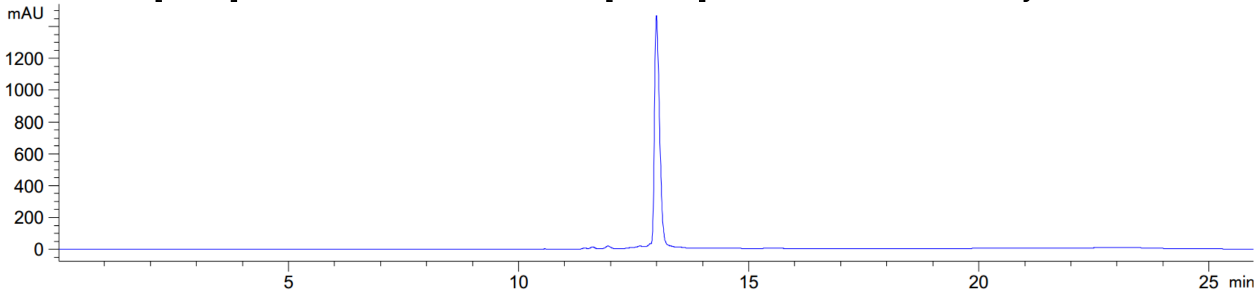

LLW 17-mer $C_{80}H_{124}N_{22}O_{18}$ retention time: 13.511 min calculated $[M+H]^{+}$: 1681.9542
Observed $[M+H]^{+}$: 1681.9519 Observed $[M+2H]^{2+}$: 841.4789 overall yield: 26%

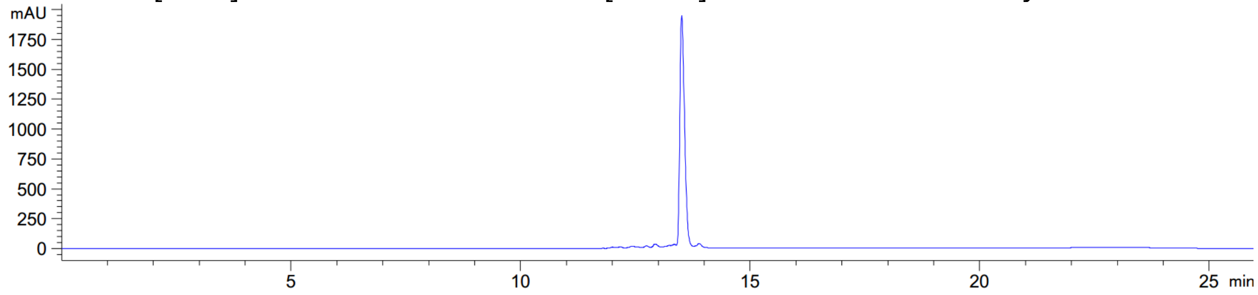

LLI 17-mer $C_{75}H_{125}N_{21}O_{18}$ retention time: 13.458 min calculated $[M+H]^{+}$: 1608.9590
Observed $[M+H]^{+}$: 1608.9558 Observed $[M+2H]^{2+}$: 804.9809 overall yield: 23%

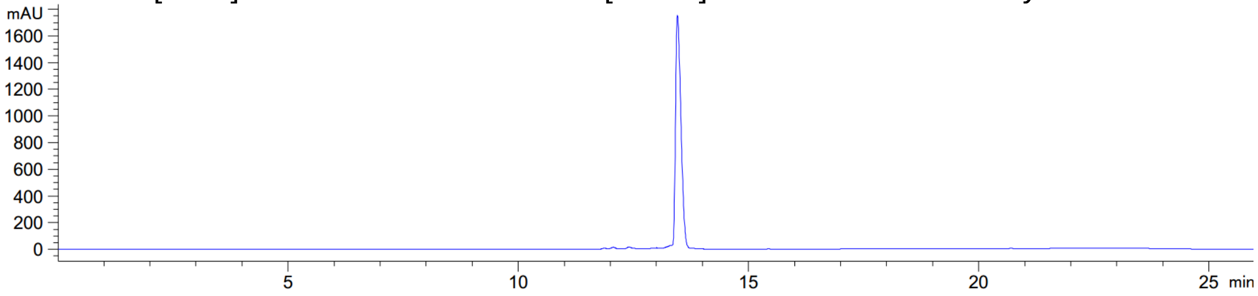

LLL 17-mer $C_{75}H_{125}N_{21}O_{18}$ retention time: 13.516 min calculated $[M+H]^{+}$: 1608.9590
Observed $[M+H]^{+}$: 1608.9555 Observed $[M+2H]^{2+}$: 804.9813 overall yield: 34%

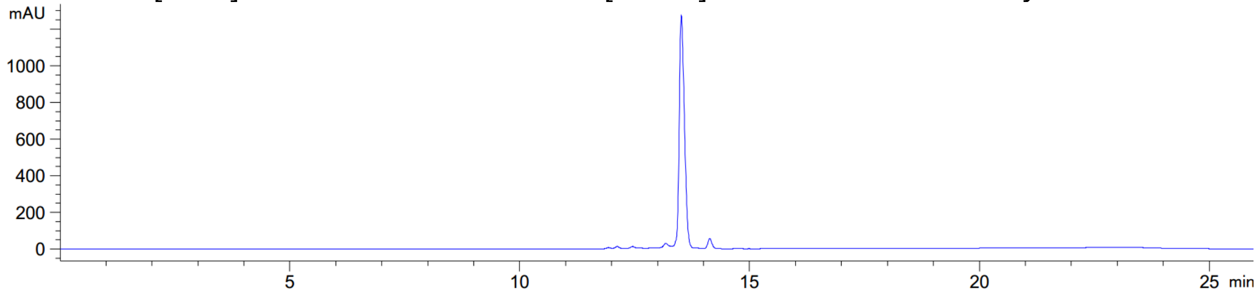

LLY 17-mer $C_{78}H_{123}N_{21}O_{19}$ retention time: 12.641 min calculated $[M+H]^+$: 1658.9382 Observed $[M+H]^+$: 1658.9362 Observed $[M+2H]^{2+}$: 829.9708 overall yield: 37%

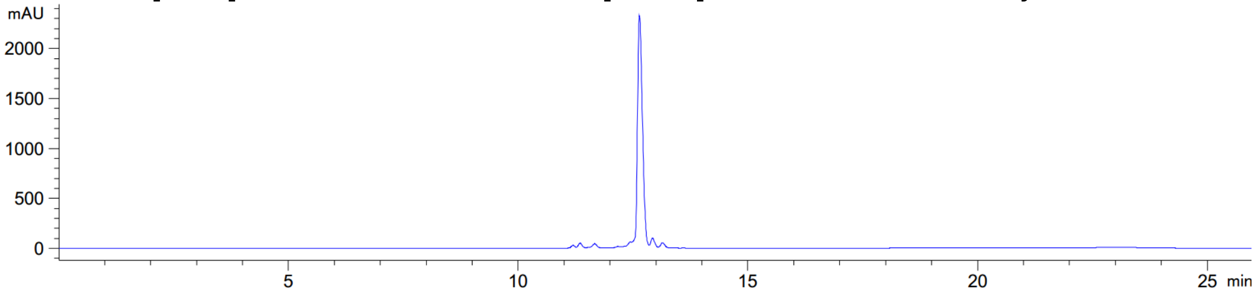

bicyclo 17-mer $C_{75}H_{113}N_{21}O_{18}S_3$ retention time: 11.908 min calculated $[M+H]^+$: 1692.7813 Observed $[M+H]^+$: 1692.7823 Observed $[M+2H]^{2+}$: 846.8930 overall yield: 24%

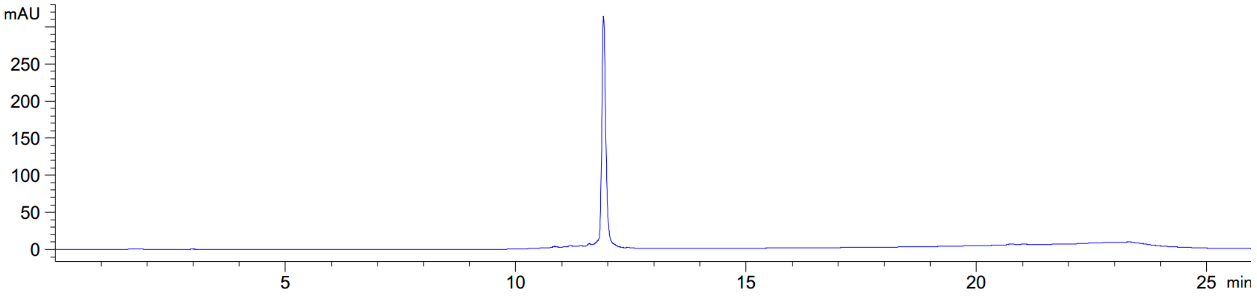

bicyclo G-A 17-mer $C_{76}H_{115}N_{21}O_{18}S_3$ retention time: 11.450 min calculated $[M+H]^+$: 1706.7969 Observed $[M+H]^+$: 1706.7978 Observed $[M+2H]^{2+}$: 853.9006 overall yield: 19%

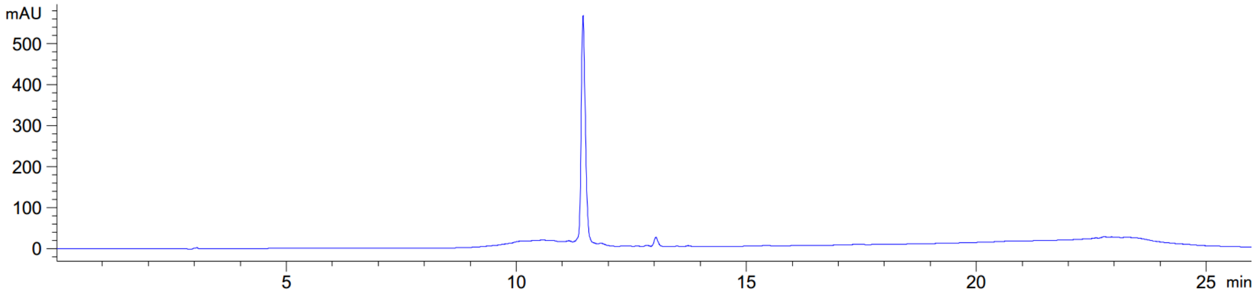

AAKA 12-mer $C_{52}H_{84}N_{16}O_{13}$ retention time: 8.389 min calculated $[M+H]^+$: 1141.6482 Observed $[M+H]^+$: 1141.6445 Observed $[M+2H]^{2+}$: 571.3263 overall yield: 22%

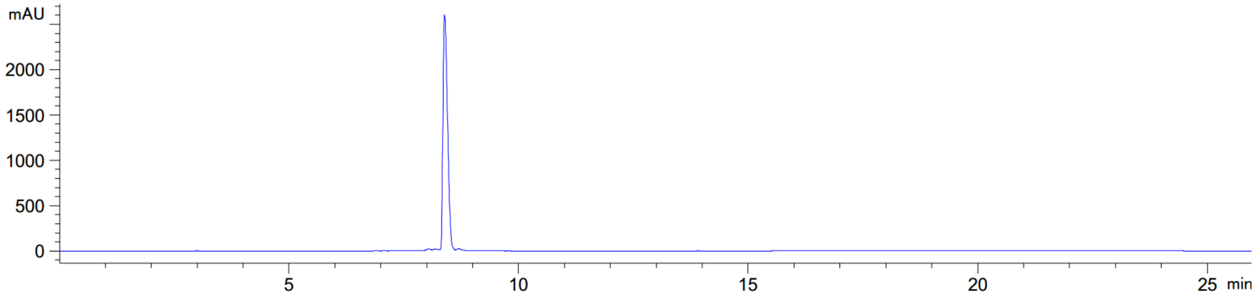

bicyclo 12-mer $C_{57}H_{81}N_{15}O_{13}S_3$ retention time: 12.115 min calculated $[M+H]^+$: 1280.5379 Observed $[M+H]^+$: 1280.5364 Observed $[M+2H]^{2+}$: 640.7713 overall yield: 18%

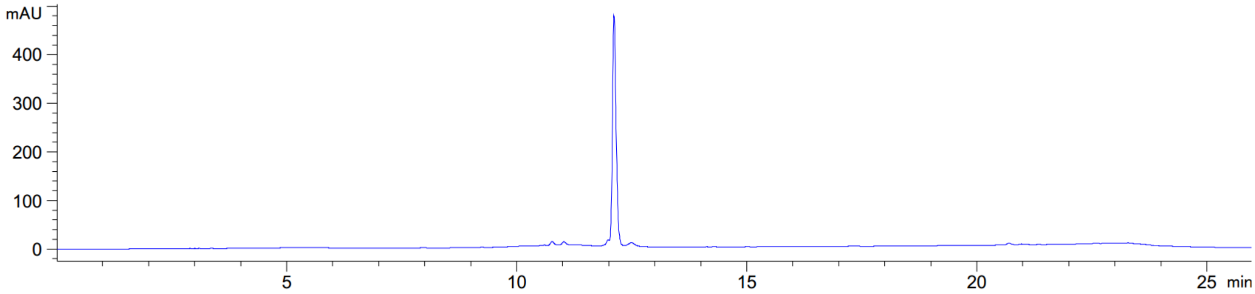

[*C3-Ccap*] 12-mer $C_{56}H_{81}N_{15}O_{13}S_2$ retention time: 11.519 min calculated $[M+H]^+$: 1236.5658 Observed $[M+H]^+$: 1236.5633 Observed $[M+2H]^{2+}$: 618.7856 overall yield: 25%

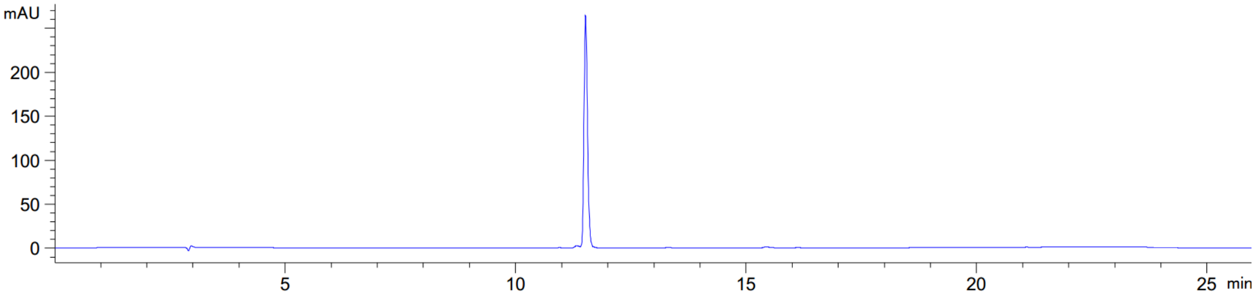

[*C3-C''*] 12-mer $C_{56}H_{81}N_{15}O_{13}S_2$ retention time: 11.580 min calculated $[M+H]^+$: 1236.5658 Observed $[M+H]^+$: 1236.5636 Observed $[M+2H]^{2+}$: 618.7856 overall yield: 21%

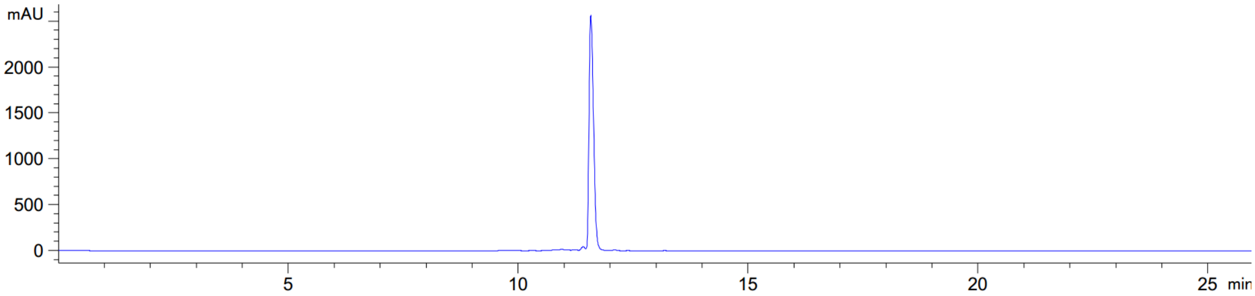

1 $C_{60}H_{103}N_{19}O_{16}$ retention time: 8.500 min calculated $[M+H]^+$: 1346.7908 Observed $[M+H]^+$: 1346.7903 Observed $[M+2H]^{2+}$: 673.8974 overall yield: 24%

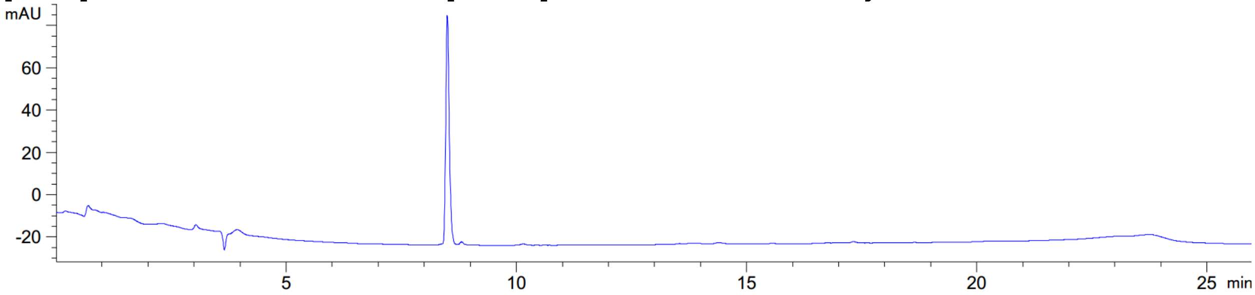

BSM-1 $C_{74}H_{115}N_{21}O_{19}S_3$ retention time: 9.656 min calculated $[M+H]^+$: 1698.7918 Observed $[M+H]^+$: 1698.7895 Observed $[M+2H]^{2+}$: 849.8973 overall yield: 18%

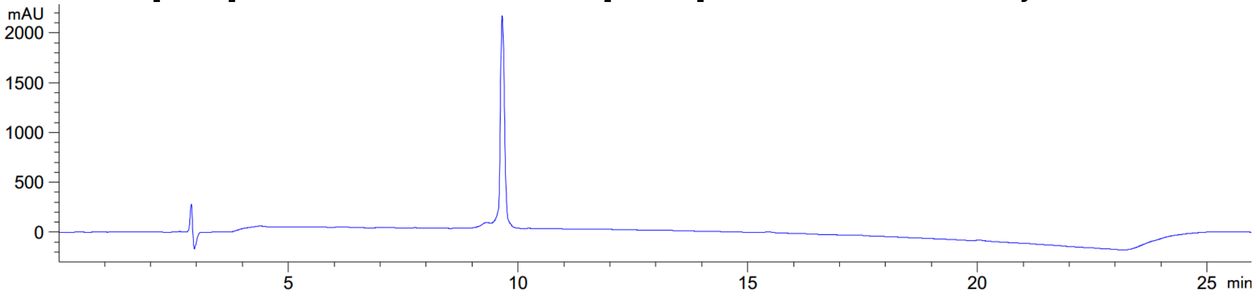

2 $C_{55}H_{90}N_{16}O_{16}$ retention time: 10.970 min calculated $[M+H]^+$: 1231.6799 Observed $[M+H]^+$: 1231.6790 overall yield: 16%

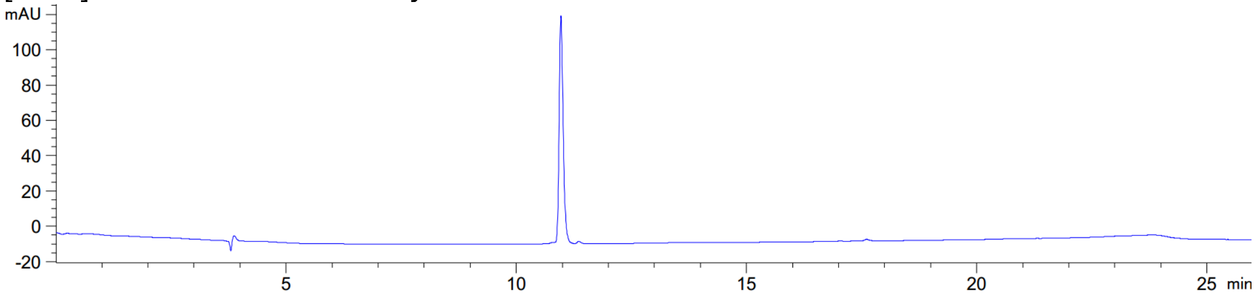

BSM-2 $C_{70}H_{107}N_{19}O_{17}S_3$ retention time: 12.170 min calculated $[M+H]^+$: 1582.7333 Observed $[M+H]^+$: 1582.7327 overall yield: 17%

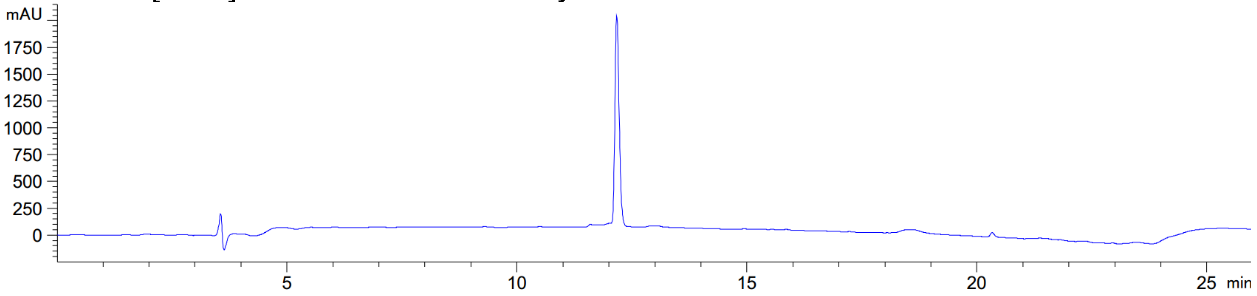

J. References

1. Tuncbag, N., Gursoy, A. & Keskin, O. Identification of computational hot spots in protein interfaces: combining solvent accessibility and inter-residue potentials improves the accuracy. *Bioinformatics* **25**, 1513-1520, doi:10.1093/bioinformatics/btp240 (2010).

2. Huang, Y., Niu, B., Gao, Y., Fu, L. & Li, W. CD-HIT Suite: a web server for clustering and comparing biological sequences. *Bioinformatics* **26**, 680-682, doi:10.1093/bioinformatics/btq003 (2010).
3. Li, W. & Godzik, A. Cd-hit: a fast program for clustering and comparing large sets of protein or nucleotide sequences. *Bioinformatics* **22**, 1658-1659, doi:10.1093/bioinformatics/btl158 (2006).
4. Kabsch, W. & Sander, C. Dictionary of protein secondary structure: pattern recognition of hydrogen-bonded and geometrical features. *Biopolymers* **22**, 2577-2637, doi:10.1002/bip.360221211 (1983).
5. Luo, P. & Baldwin, R. Mechanism of helix induction by trifluoroethanol: a framework for extrapolating the helix-forming properties of peptides from trifluoroethanol/water mixtures back to water. *Biochemistry* 36, 8413–8421, doi:10.1021/bi9707133 (1997).
6. Rohl, C & Baldwin, R. Deciphering rules of helix stability in peptides. *Methods in Enzymology* **295**, 1-26, doi:10.1016/S0076-6879(98)95032-7 (2004).
7. Zerbe, O. & Bader, R. Peptide NMR. *https://www.chem.uzh.ch/zerbe/PeptidNMR.pdf*
8. Ianders, G., Alogheli, H., Brandt, P. & Karlén, A. Conformational analysis of macrocycles: comparing general and specialized methods. *J. Comput. Aided Mol. Des.* **34**, 231-252, doi:10.1007/s10822-020-00277-2 (2020).
9. Nguyen, D., Mayne, L., Phillips, M. C. & Walter Englander, S. Reference Parameters for Protein Hydrogen Exchange Rates. *J. Am. Soc. Mass Spectrom* **29**, 1936-1939, doi:10.1007/s13361-018-2021-z (2018).
